# Identification of cuproptosis and immune-related gene prognostic signature in lung adenocarcinoma

**DOI:** 10.3389/fimmu.2023.1179742

**Published:** 2023-08-09

**Authors:** Wentao Zhang, Haizeng Qu, Xiaoqing Ma, Liang Li, Yanjun Wei, Ye Wang, Renya Zeng, Yuanliu Nie, Chenggui Zhang, Ke Yin, Fengge Zhou, Zhe Yang

**Affiliations:** ^1^ Tumor Research and Therapy Center, Shandong Provincial Hospital Affiliated to Shandong First Medical University, Jinan, Shandong, China; ^2^ Radiotherapy Department, Dongming People’s Hospital, Heze, Shandong, China; ^3^ Radiotherapy and Minimally Invasive Group I, The Second Affiliated Hospital of Shandong First Medical University, Taian, Shandong, China; ^4^ Department of Thoracic Surgery, Shandong Provincial Hospital, Cheeloo College of Medicine, Shandong University, Shandong, China; ^5^ Department of Radiation Oncology, Weifang People's Hospital, Weifang, China; ^6^ Tumor Research and Therapy Center, Shandong Provincial Hospital, Cheeloo College of Medicine, Shandong University, Jinan, Shandong, China; ^7^ Department of Orthopedics, Shandong Provincial Hospital Affiliated to Shandong First Medical University, Jinan, Shandong, China; ^8^ Department of Pathology, Shandong Provincial Hospital, Cheeloo College of Medicine, Shandong University, Jinan, Shandong, China

**Keywords:** cuproptosis, immune, LUAD, prognosis, signature

## Abstract

**Background:**

Cuproptosis is a novel form of programmed cell death that differs from other types such as pyroptosis, ferroptosis, and autophagy. It is a promising new target for cancer therapy. Additionally, immune-related genes play a crucial role in cancer progression and patient prognosis. Therefore, our study aimed to create a survival prediction model for lung adenocarcinoma patients based on cuproptosis and immune-related genes. This model can be utilized to enhance personalized treatment for patients.

**Methods:**

RNA sequencing (RNA-seq) data of lung adenocarcinoma (LUAD) patients were collected from The Cancer Genome Atlas (TCGA) and Gene Expression Omnibus (GEO) databases. The levels of immune cell infiltration in the GSE68465 cohort were determined using gene set variation analysis (GSVA), and immune-related genes (IRGs) were identified using weighted gene coexpression network analysis (WGCNA). Additionally, cuproptosis-related genes (CRGs) were identified using unsupervised clustering. Univariate COX regression analysis and least absolute shrinkage selection operator (LASSO) regression analysis were performed to develop a risk prognostic model for cuproptosis and immune-related genes (CIRGs), which was subsequently validated. Various algorithms were utilized to explore the relationship between risk scores and immune infiltration levels, and model genes were analyzed based on single-cell sequencing. Finally, the expression of signature genes was confirmed through quantitative real-time PCR (qRT-PCR), immunohistochemistry (IHC), and Western blotting (WB).

**Results:**

We have identified 5 Oncogenic Driver Genes namely CD79B, PEBP1, PTK2B, STXBP1, and ZNF671, and developed proportional hazards regression models. The results of the study indicate significantly reduced survival rates in both the training and validation sets among the high-risk group. Additionally, the high-risk group displayed lower levels of immune cell infiltration and expression of immune checkpoint compared to the low-risk group.

## Introduction

Lung cancer (LC) is a prevalent form of cancer worldwide, and the number of cases is rising each year. Lung adenocarcinoma (LUAD) is the most common subtype of lung cancer, accounting for approximately half of all cases (1). Although there have been advances in cancer treatment, LUAD patients’ treatment outcomes are still unsatisfactory due to metastasis and recurrence. Therefore, there is an urgent need for new prognostic markers to evaluate patients’ prognosis and guide treatment decisions.

Copper is an essential trace element in the human body (2), but excess copper ions in cells can trigger a new form of cell death called cuproptosis (3–5). During this process, copper ions bind directly to fatty acylation components in the tricarboxylic acid cycle (TCA) in mitochondrial respiration, leading to the aggregation of fatty acylated proteins and the loss of iron-sulfur cluster proteins (6). This causes proteotoxic stress and ultimately results in cell death. Additionally, the tumor microenvironment (TME) (7–9) is composed of immune cells, stromal cells, extracellular matrix, and peripheral blood vessels, all of which have a significant impact on tumor growth, metabolism, and metastasis. Among these, immune cells play a crucial role (10, 11). Studies have shown that infiltrating immune cells in LUAD are closely related to tumor aggressiveness and patient prognosis (12, 13).

It is worth noting that there is a connection between copper and immunity. Previous studies have shown that copper is essential for the development and maintenance of the immune system. Copper deficiency can lead to a reduction in immune cells (14). In the immune system, T cells and B cells are crucial components (15). Copper deficiency may hinder the development of T cells and affect their function. Some studies have suggested that copper can also affect the proliferation and activity of T cells (16, 17). Copper deficiency can lead to a decreased ability of the human immune system to respond to various diseases and infections. In addition, copper is also critical for the function of B cells (18). Copper deficiency can affect the ability of B cells to secrete immunoglobulin, thereby reducing the body’s protection against pathogens (18). Interleukin-2 is also an important immune molecule in the immune system and is one of the important factors that activate T cells (18). However, in the case of copper deficiency, the production of interleukin-2 is suppressed, affecting the activation ability of T cells, thus leading to a decrease in the body’s immune response to pathogens, which makes it susceptible to various infections and diseases. Moreover, copper also participates in the synthesis of a large number of antioxidant enzymes, including superoxide dismutase and glutathione peroxidase (19). These enzymes have the function of clearing free radicals in the body, protecting immune cells from oxidation damage, and enhancing the body’s resistance (19). Recent studies have also found that copper can affect the expression of PD-L1 in cancer cells, which is a key signaling pathway for immune evasion (20). Overall, copper plays a very important role in the immune system.In this study, we aim to investigate the value of cuproptosis and immune-related genes (CIRGs) on the prognosis and immunotherapy of LUAD patients through innovative bioinformatics methods. By examining this relationship, we can improve personalized treatment for patients.

## Materials and methods

### Data resources

This study obtained RNA sequencing (RNA-seq) data, clinical data, single cell sequencing data, and simple nucleotide variation data from the Genomic Data Commons (GDC) and The Cancer Genome Atlas (TCGA) (https://portal.gdc.cancer.gov/). RNA-seq data and clinical data were also obtained from the National Center for Biotechnology Information (NCBI) Gene Expression Omnibus (GEO) database (https://www.ncbi.nlm.nih.gov/geo/, ID: GSE68465, GSE72094, GSE37745). Additionally, 19 cuproptosis-related genes were obtained from literature sources.

### Identification of genes associated with cuproptosis and immune

First, we cleaned up missing values in the RNA-seq and clinical data. We selected GSE68465 as the training set and used the “GSVA” R package to perform single sample gene set enrichment analysis (ssGSEA) on all samples in the training set, obtaining scores for immune cell infiltration and immune function, including aDCs, APC co-inhibition, APC co-stimulation, B cells, CCR, CD8+ T cells, Check-point, Cytolytic activity, DCs, HLA, iDCs, Inflammation-promoting, Macrophages, Mast cells, MHC class I, Neutrophils, NK cells, Parainflammation, pDCs, T cell co-inhibition, T cell co-stimulation, T helper cells, Tfh, Th1 cells, Th2 cells, TIL, Treg, Type I IFN Response, Type II IFN Response. Based on the results of ssGSEA (21) on the training set, we used weighted gene co-expression network analysis (WGCNA) (22) to screen for immune-related genes. We used the “PickSoftThreshold” function to automatically select a soft threshold value and performed scale-free and average connectivity analysis on modules with different power values. Then, we obtained corresponding dissimilarity matrix (1-TOM) and topological overlap matrix (TOM). We performed Pearson correlation analysis on the co-expression modules based on ssGSEA scores. The module with the highest correlation with immune indicators was selected as the immune-related genes (IRGs) screened by WGCNA. To validate the WGCNA-screened IRGs, we used the “clusterProfiler” and “enrichplot” R packages for KEGG (Kyoto Encyclopedia of Genes and Genomes) and GO (Gene Ontology) enrichment analysis to demonstrate the relationship between IRGs and immunity.

Based on the results of “ConsensusClusterPlus” R package and ssGSEA, we performed consensus clustering analysis on the samples in GSE68465. We increased the clustering variable (K) from 2 to 10 and found the optimal K value, which provided the highest intra-cluster correlation and the lowest inter-cluster correlation. We used the “survival” and “survminer” R packages for Kaplan-Meier (KM) analysis of the CRG-related clusters to compare differences in overall survival (OS). Then, we used the “DeSeq2” R package for differential analysis of clustering (|log2FC|≥1 and FDR<0.05), and the analysis result was new IRGs. Subsequently, we used ssGSEA, Cibersort, and Estimate algorithms to obtain immune scores to validate the effectiveness of immune clustering.

Next, we obtained 18 cuproptosis genes from the literature and also performed unsupervised clustering to divide them into two clusters. We then performed survival, clinical, and immune-related analysis on the cuproptosis related clusters. The intersection of the genes selected by the above three methods is the CIRGs.

### Establishment and validation of CIRG prognostic model

Univariate Cox analysis was performed on CIRGs to screen out genes related to survival with statistical significance (P<0.05). Five machine learning algorithms, including decision trees, random forests, LASSO, GBDT, and XGBoost, were used to evaluate the weights of CIRGs related to survival and calculate their average values. The top ten genes were selected to construct a LASSO Cox model using the R package “glmnet”. The best penalty coefficient (λ) was selected using ten-fold cross-validation. All samples were divided into high and low-risk groups based on the median value of the risk score in the training set. The high-risk and low-risk groups were analyzed using KM analysis, and the accuracy of the model was evaluated using ROC analysis with the R package “timeROC”. Additionally, a heatmap was used to show the differences in T stage, N stage, sex, and age between the high-risk and low-risk groups in terms of risk score and clinical information. Finally, a nomogram combining risk scores and clinical data was constructed, and correction curves were plotted.

### Analysis of survival and immune infiltration of OCIRGs

We performed a series of analyses on OCIRGs, which are core genes used to build our model. First, we selected the optimal survival-related cut-off value for OCIRG expression using the R packages “survminer” and “survival”, and analyzed the difference in survival between patients with high and low expression of OCIRG. Additionally, we batch-corrected and combined RNA-seq data from the GSE68465 and TCGA LUAD cohorts, and analyzed whether OCIRG expression differed between tumor and normal tissues. We also used CIBERSORT (23)., a tool for analyzing immune cell infiltration levels through gene expression profiling assessment, to evaluate the correlation between the expression levels of OCIRGs and the infiltration levels of various immune cells.

### Enrichment analysis related to pathway and function

GSEA (Gene Set Enrichment Analysis) and GSVA (Gene Set Variation Analysis) (24) are important tools for enrichment analysis. In this study, both methods were used to analyze the pathways and functions associated with the risk model. KEGG (Kyoto Encyclopedia of Genes and Genomes) enrichment analysis was performed using GSEA software (version 4.2.3). GO enrichment analysis evaluates gene molecular function, cellular components, and biological processes at three levels. Additionally, GSVA was used to analyze the correlation between risk score and popular pathways, including Hippo, Wnt, MAPK, PI3K/AKT, TGF-β, NF-kB, Notch, AMPK, JAK-STAT, PD-1/PD-L1, mTOR, Ras, TNF, HIF-1, and ErbB.

### Exploration of tumor immune microenvironment

In this study, we explored the role of the tumor immune microenvironment in cancer by evaluating the level of immune cell infiltration using various algorithms from TIMER2.0, such as TIMER, CIBERSORT, QUANTISEQ, MCPCOUNTER, XCELL, and EPIC. We analyzed the differences in immune cell infiltration between high-risk and low-risk groups. In addition, we used ssGSEA to evaluate immune cell infiltration levels and immune function. We also examined the expression levels of immune checkpoints and analyzed differences between the high-risk and low-risk groups

Furthermore, we used the ESTIMATE algorithm to assess the relationship between tumor purity and risk scores, including estimated score, immune score, and stromal score. It is important to note that the tumor immune microenvironment plays a crucial role in cancer development and treatment. By analyzing immune cell infiltration levels and immune function, we can better understand the mechanisms underlying cancer and potentially develop new treatment strategies.

### Analysis of associations between risk subtypes and mutational landscapes

We obtained single nucleotide polymorphism (SNP) data of LUAD from the TCGA database. Using MAFTOOL software, we displayed the top-ranked mutated gene maps in the high-risk and low-risk groups, along with their mutation types and frequencies, and assessed the correlation between mutation counts and risk scores. Additionally, we analyzed the tumor mutational burden (TMB) in the high-risk and low-risk populations.

### Characterization of OCIRGs by single-cell RNA sequencing

Single-cell RNA-seq data of 15 LUAD samples were obtained from GSE131907 in the GEO database. The sequencing data was analyzed based on the “Seurat” R package, and high-quality cells were screened out using the “CreateSeuratObject” function, where “PercentageFeatureSet” was used to calculate the percentage of mitochondrial genes in each cell. Quality control was performed according to the following criteria: retained genes were expressed in at least 3 cells; cells with less than 50 gene expression were eliminated; the percentage of ribosomal genes was less than 20%. Normalize the filtered data using the “LogNormalize” method in the “NormalizeData” function. “FindVariableFeature” was used to identify highly variable genes, followed by principal component analysis (PCA) using the “RunPCA” function to reduce the dimensionality of the single-cell sequencing data based on the top 1500 genes. The “jackstraw” function identified important PCs, and the top 20 PCs were selected for cell clustering analysis using a distributed stochastic neighborhood embedding (t-SNE) algorithm. Cell clusters were tool-annotated using the “FindAllMarkers” function to calculate the DEG for each cluster.

### Immunohistochemistry validation of the protein expression levels of OCFRGs

Five Lung adenocarcinoma tissue chips were purchased from Shanghai Outdo Biotech Company (Shanghai, China). Each tissue chip includes 45 cancer tissues and 45 paracancerous tissues. CD79B (rabbit polyclonal, catalog number: ab134147, Abcam), PEBP1 (rabbit polyclonal, catalog number: ab76582, Abcam), PTK2B (rabbit polyclonal, catalog number: ab32571, Abcam), STXBP1 (rabbit polyclonal, catalog number: ab124920, Abcam): ab126512, Abcam) and ZNF671 (rabbit polyclonal, catalog number: JP39176, Product Datasheet). The results of the immunohistochemical staining were scored. Semiquantitative scoring was performed according to the staining intensity and the percentage of positive cells: No staining, pale yellow (light yellow particles), medium (brown yellow particles), and heavy (dark brown particles) were scored as 0, 1, 2, and 3, respectively. According to the percentage of positively stained cells in the total number of cells, 0% was scored as 0, 5% to 25% was scored as 1; 26% to 50% was scored as 2; 51% to 75% was scored as 3; and >75% was scored as 4 points. The final score was the sum of the staining intensity and the percentage of positive cells. The sum of the staining intensity and the percentage of positive cells was less than 6 for the low expression group and ≥ 6 for the high expression group. Five 400x high-power fields were randomly selected for each section, the staining intensity and percentage of positive cells were scored in each field, and the average value was calculated. The immunohistochemical staining results were microscopically adjudicated by two pathologists in an independent, double-blind manner.

### Quantitative real-time polymerase chain reaction (qRT-PCR)

Normal lung epithelial cells BEAS-2B cells and four human lung adenocarcinoma cell lines A549, H1299, PC9, and H23 were obtained from the Central Laboratory of Shandong Provincial Hospital. PCR ARRAY was obtained from Shanghai Outdo Biotech Company (Shanghai, China). Total RNA was extracted using TRIzol reagent (Invitrogen, USA). Complementary DNA (cDNA) was synthesized using the PrimeScript RT kit (Takara).

### Western blotting

Cells were lysed in cold Radioimmunoprecipitation assay (RIPA) buffer. The same amount of protein was subjected to SDS-PAGE, and then transferred to PVDF (polyvinylidene fluoride) membrane. Block with nonfat dry milk containing TBST for 1 h. The primary antibody (western blot and IHC universal primary antibody) was diluted according to the instructions and incubated overnight at 4°C. After washing with TBST, the secondary antibody was added and incubated for 1 hour at room temperature. After washing the membrane, it was developed using enhanced chemiluminescence (ECL) chromogenic solution.

### Transfection of cell lines

CD79B specific siRNA and negative control siRNA for human were purchased from RiboBio Co. Ltd. (Guangzhou, China). The siRNA sequences are shown in **Supplementary Table S2**. RT-qPCR analysis was performed 72 hours post-transfection to examine the transfection efficiency.

### CCK-8 assay

Cell proliferation was measured using the CCK-8 assay. A549 and H1299 cells were seeded in 96-well plates, cultured for 0, 24, 48, and 72 h, and incubated with CCK-8 solution for 1h in the dark. Absorbance values were measured at 450 nm using a microplate reader.

### Colony formation assay

A549 and H1299 cells were cultured in 6-well tissue culture plates for 1 week until colonies were formed. Then, the cell colonies were fixed with 0.5% polyformaldehyde (Servicebio, Beijing, China) for 25 minutes and stained using 2.5% methylene violet dye for 15 minutes. After washing, the cell colonies were recorded and counted.

### Wound healing assay

In the wound-healing assay, 24-well plates were used to seed cells. Using a sterile tip, cells were scratched perpendicular to the previously painted line. After imaging the scratch wounds with a light microscope, cell migration was measured at time points of 0 and 24 h.

### Transwell assay

Transwell assays were conducted with 24-well transwell chambers to evaluate the cellular invasiveness of A549 and H1299 cell lines. The cells were introduced into upper chambers either with or without Matrigel in serum-free culture medium. The lower chambers were supplied with 10% serum-containing culture medium (600 μl). After 24 hours, the cells were immobilized and stained.

### Statistical analysis

All statistical tests and bioinformatics analyzes in this study were completed using R (for version 4.0.1) and GSEA software (for version 4.2.3). These include the Wilcoxon rank sum test, Pearson chi-square test, T test, and logarithmic sum test. p<0.05 was considered statistically significant.

## Results

### Identification of cuproptosis/immune-related genes

**Figure 1:**
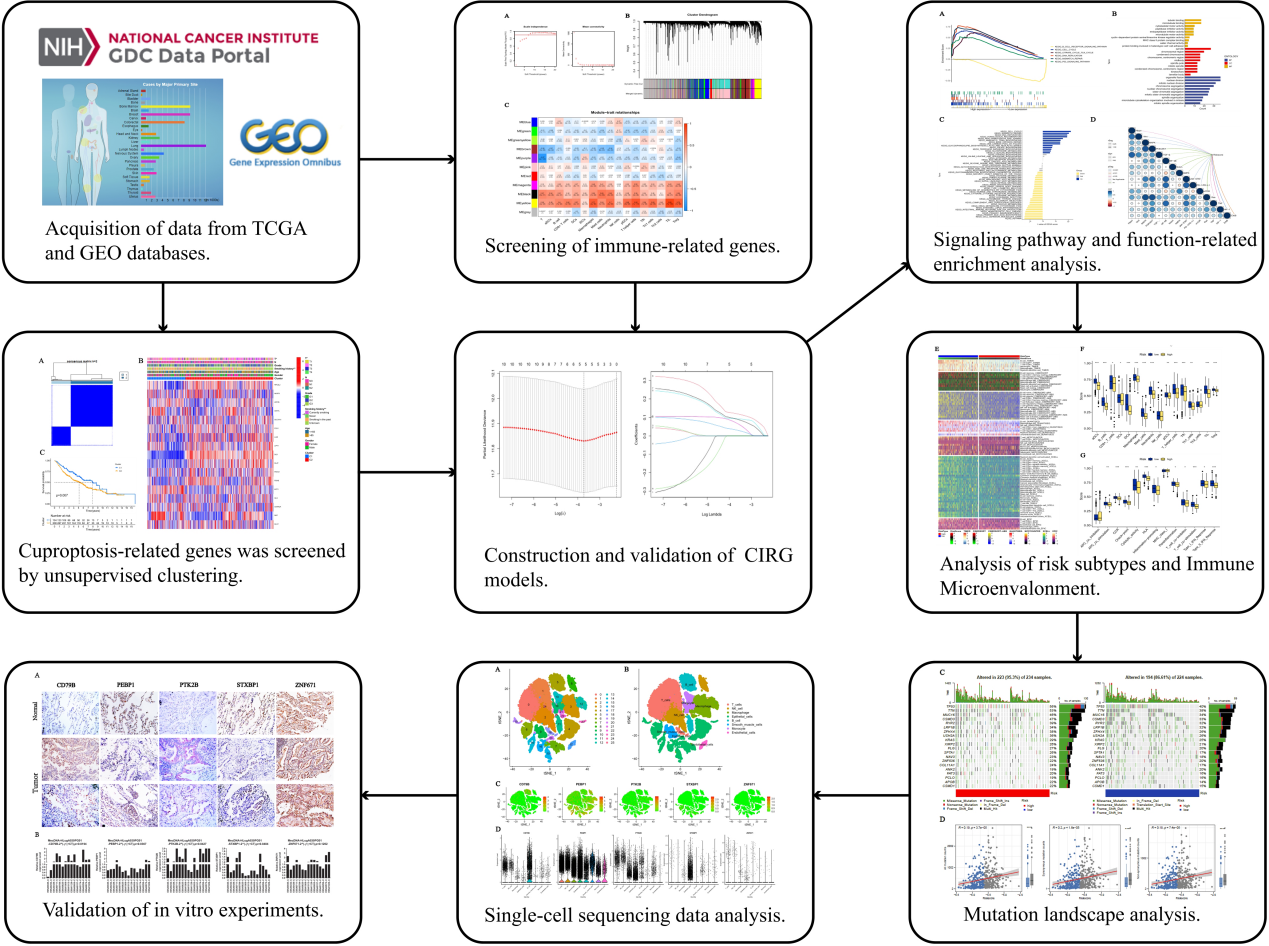
Work flow of the study. This figure shows the construction process and subsequent analysis of the CIRG model. *P<0.05, **P<0.01, ***P<0.001.

**Figure 2:**
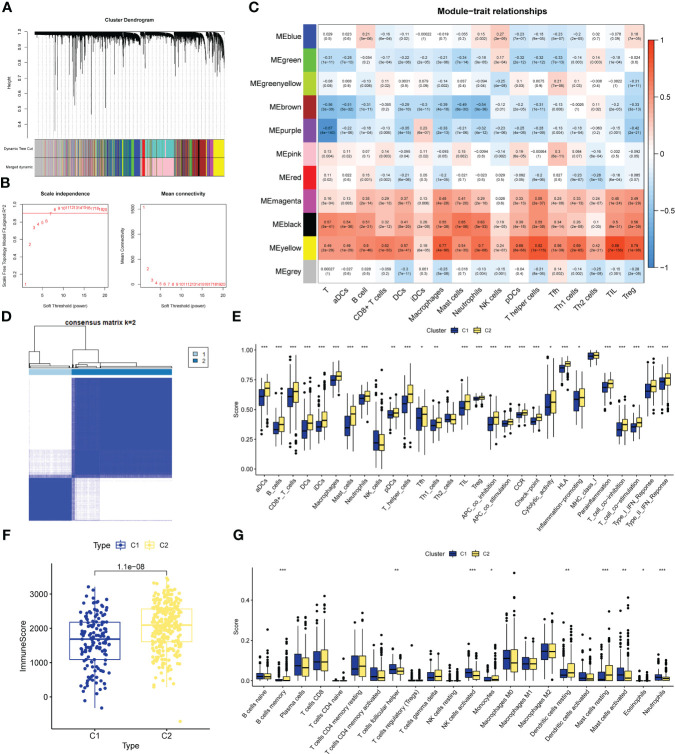
CIRG were screened by WGCNA. **(A)** The distribution and trends of the scale free topology model fit and meanconnectivity along with soft threshold. **(B)** The clustering of genes among different modules by the dynamic trees cut andmerged dynamic method. The gray modules represent unclassified genes. **(C)** Average correlation between multiplemodules and tumor development, levels of immune cell infiltration. The color of the cell indicates the strength of thecorrelation and the number in parentheses indicates the P-value for the correlation test. **(D)** Consensus clustering matrix with K-2:443 lung adenocarcinoma patient were divided into two cuproptosis-related cluster. **(E–G)** Different sis of immune fractions in SSGSEA, Estimate. Cibersort in immune-related clusters. *P<0.05, **P<0.01, ***P<0.001.

The specific process of this study is shown in **Figure 1**. First we perform WGCNA based on the results of the training set ssGSEA. Use the “pickSoftThreshold” function in the “WGCNA” R package to automatically select a soft threshold of 7 (**Figure 2A**). Multiple gene modules were divided by a dynamic cutting method, and then all modules were clustered using the “mergeCloseModules” function to obtain the final module (**Figure 2B**). We used Pearson correlation analysis and selected the most correlated module as “yellow” (**Figure 2C**), which contained a total of 1090 immune-related genes. To prove the correlation between “yellow” module genes and immunity, we used KEGG and GO databases to conduct enrichment analysis on them. KEGG results show that the “yellow” module genes are mainly enriched in Primary immunodeficiency, Antigen processing and presentation, Cell adhesion molecules, Chemokine signaling pathway, Phagosome, Intestinal immune network for IgA production, Inflammatory bowel disease, B cell receptor signaling pathway, Th17 cell Differentiation, NF−kappa B signaling pathway, Natural killer cell mediated cytotoxicity, Th1 and Th2 cell differentiation, Cytokine−cytokine receptor interaction and other functions and pathways (**Figure S1**). The results of GO are positive regulation of leukocyte activation, regulation of leukocyte cell−cell adhesion, regulation of T cell activation, mononuclear cell differentiation, leukocyte mediated immunity, leukocyte cell−cell adhesion, immune response−regulating signaling pathway, T cell activation MHC protein complex, MHC class II protein complex binding, MHC protein complex binding, cytokine receptor activity, immune receptor activity (**Figure S2**).

We performed unsupervised clustering of the 443 LUAD samples in GSE68465 to obtain subgroup types associated with the ssGSEA data. Evaluate the cluster value (K) is 2-10 results. The results showed that when K = 2, the within-group relationship was strongest and the cluster stability of each group was the best (**Figure 2D**). Furthermore, to further explore immune-related clusters, we validated them with various immune-related algorithms. In the ssGSEA algorithm, cluster2 is in aDCs, B cells, CD8+ T cells, DCs, iDCs, Macrophages, Mast cells, Neutrophils, pDCs, T helper cells, Tfh, Th1 cells, TIL, Treg, APC co inhibition, APC co stimulation, The scores of CCR, Check-point, Cytolytic activity, HLA, Inflammation-promoting, Parainflammation, T cell co-inhibition, T cell co-stimulation, Type I IFN Reponse, and Type II IFN Reponse were all higher than cluster1 (**Figure 2E**), There is a statistical difference (p<0.05). In the Estimate algorithm, the immune score of cluster2 was higher than that of cluster1 (**Figure 2F**), which was statistically significant (p<0.001). In addition, in the Cibersort algorithm, the infiltration levels of memory B cells, monocytes, resting dendritic cells, and resting mast cells in cluster C2 were higher than those in C1, and there was a statistically significant difference (p<0.05, **Figure 2G**). Differential analysis was performed on the clusters related to IRG, and 5098 new IRGs were screened (|logFC|≥1, p<0.05).

**Figure 3:**
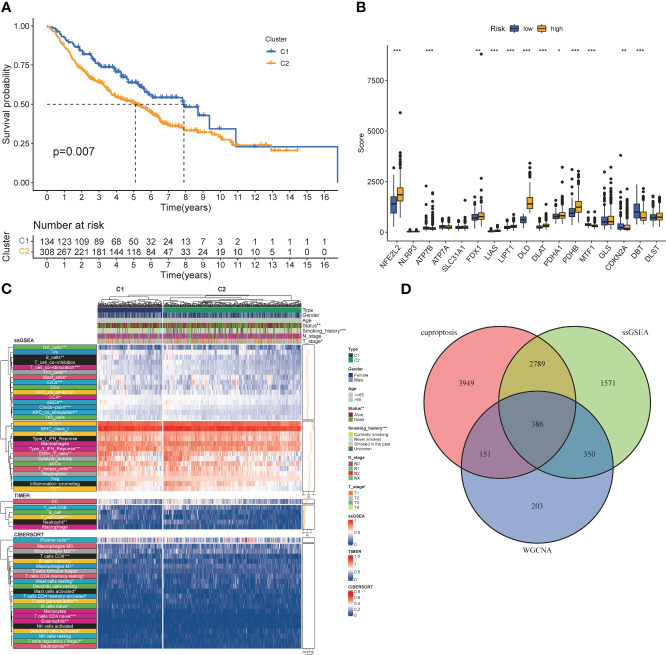
Co-screening ofCIRGS by WGCNA and cuproptosis clustering. **(A)** Kaplan-Meier survival curves for patients in the two clusters. **(B)** Differences in the expression of cuprotosis-related genes between the two clusters. **(C)** CRGs-related clusters differed in the abundance ofimmune cell infiltrates. **(D)** Through WGCNA SSGSEA. unsupervised clustering and other algorithms, a total of 386 CIRGs was obtained. *P<0.05, **P<0.01, ***P<0.001.

Screening of CRGs is still obtained by unsupervised clustering. At this time, the stability of clustering is the best when K=2 (**Figure S3**). The copper death-associated clusters differed in survival (**Figure 3A**), and the expression of copper death genes differed between the two clusters (**Figure 3B**). CRGs-related clusters differed in the abundance of immune cell infiltrates (**Figure 3C**). Differential analysis was performed on the clusters related to CRGs, and 7275 CRGs were screened out (|logFC|≥1, p<0.05). A total of 386 CIRGs were obtained by intersecting the genes screened by the above method (**Figure 3D**).

### Development of the CIRG model

**Figure 4:**
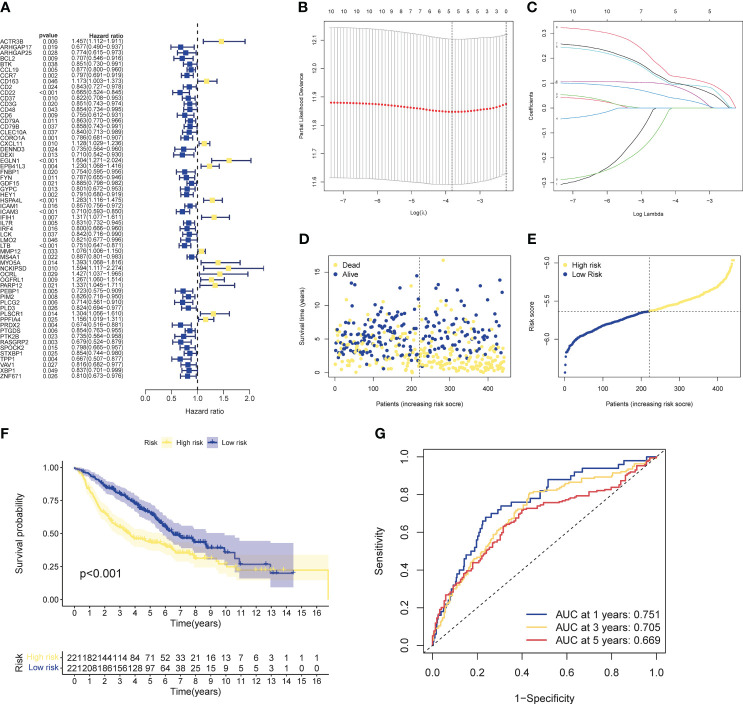
Development of risk profiles in LUAD patients collected from the GEO cohort GSE68465. **(A)** Univariate Coxregression of 44 GIRGs in LUAD. **(B)** LASSO regression of the top 10 CIRGs with survival weights screened by machine learning. **(C)** Cross-validation in the LASSO regression for optimizing parameter selection. **(D)** Distribution of LUAD patients based on risk scores. **(E)** Distributions of OS status, OS and risk scores. **(F)** KM curves for OS of LUAD patients in different clusters. **(G)** ROC curves of this signature.

Univariate cox analysis was used to evaluate CIRGs associated with survival (**Figure 4A**). Based on five machine learning algorithms, the weights associated with CIRG survival were evaluated and the top ten genes (**Table 1**) were used to construct the LASSO cox model (**Figures 4B, C**). The risk score is calculated as follows:

**Table 1:** The quantified importance of prognostic cuproptosis/immune-related messenger genes by machin learning.

	Decision Tree	LASSO	Random Forest	GBDT	XGBoost	AVG	
CD163	0.032805	0.212120	0.055783	0.118056	0.026271	0.089007	(1)
CD79B	0.018490	0.095986	0.032053	0.072803	0.016091	0.047084	(2)
STXBP1	0.020378	0	0.065696	0.088047	0.026053	0.040034	(3)
ZNF671	0.015497	0.090091	0.038642	0.027597	0.011142	0.036594	(4)
PEBP1	0.052572	0.032980	0.027951	0.049932	0.012401	0.035167	(5)
GDF15	0.024313	0.034979	0.058619	0.030896	0.023375	0.034437	(6)
PTK2B	0.009227	0.112742	0.017402	0.014422	0.013358	0.033430	(7)
ACTR3B	0.019116	0.060720	0.014346	0.045244	0.014739	0.030833	(8)
ATXN1	0	0.041379	0.038957	0.032762	0.023866	0.027393	(9)
LY9	0.026432	0.047058	0.009882	0.027525	0.023154	0.026810	(10)

The number in the parentheses represented the rankings of weight.


$$\begin{array}{c} \text{Risk score}=\left(-0.1016*\text{CD}79\text{B exp.}\right)+\left(-0.2292*\text{PEBP}1\text{ exp.}\right)\\ +\left(-0.2188\;*\text{ PTK}2\text{B exp.}\right)\\ +\left(-0.1128\;*\text{ STXBP}1\text{ exp.}\right)\\ +\left(-0.1204\;*\text{ ZNF}671\text{ exp.}\right) \end{array}$$

The median risk score of the training set samples was used as a cutoff value to divide all patients into high-risk and low-risk groups (**Figure 4D**). High-risk patients had significantly shorter survival time and higher mortality (**Figure 4E**), and the high-risk and low-risk groups were well separated (**Figure S4-5**). KM analysis showed that there was a significant difference in survival between the high-risk and low-risk groups in the training set (p<0.001), and the survival of high-risk patients was significantly shortened (**Figure 4F**). Furthermore, from the results of the ROC analysis, the AUC values at 1 year, 3 years, and 5 years were 0.751, 0.705, and 0.669, respectively (**Figure 4G**). Through cox analysis, we found that age (HR=1.032, 95% confidence interval (CI)=1.018-1.046, p<0.001), T stage (HR=1.955, 95% confidence interval (CI)=1.170-1.712, p < 0.001), N stage (HR=1.415, 95% confidence interval (CI)=1.658-2.304, p<0.001), risk score (HR=2.656, 95% confidence interval (CI)=1.609-4.386, p<0.001) is an independent prognostic factor (**Table 2**).

**Table 2:** Independent analysis of training set patients.

Characteristics	Univariate			Multivariate		
	HR	95%CI	P	HR	95%CI	P
Age	1.026	1.013-1.040	<0.001	1.032	1.018-1.046	<0.001
T stage	1.654	1.376-1.987	<0.001	1.955	1.170-1.712	<0.001
N stage	1.993	1.694-2.344	<0.001	1.415	1.658-2.304	<0.001
Risk score	3.020	2.013-5.071	<0.001	2.656	1.609-4.386	<0.001

HR, hazard ratio; CI, confidence interval.

### Validation of prognostic models and analysis of clinical characteristics

**Figure 5:**
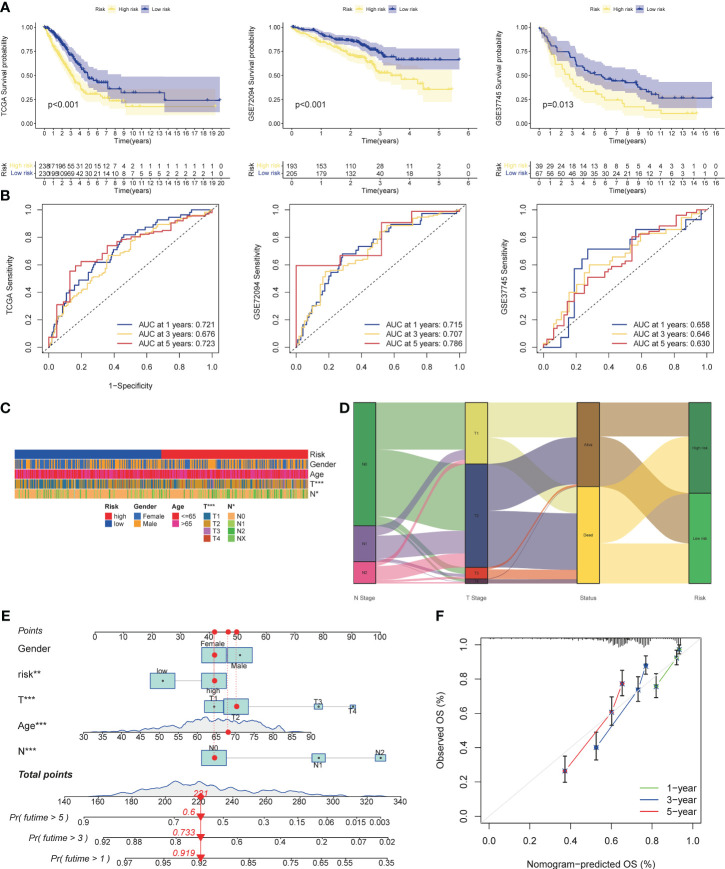
Validation of risk models, prognostic clinical value and nomogram. **(A)** Survival curves of high and low risk group in the validation set (TCGA cohort, GSE37745). **(B)** AUC values of ROC curves for risk scores in the validation set (TCGA cohort, GSE72094, GSE37745). **(C)** Different stratification of clinical phenotypes in the high- and – low-risk groups. **(D)** Connection among the risk subtypes, vital status, T stage and N stage stratification. **(E)** Nomogram for 1-3,and 5-years overall survival prediction. The red line show an example of how to predict the prognosis. **(F)** Calibration plots for agreement tests between predicted and actual OS. *P<0.05, **P<0.01, ***P<0.001.

Three cohorts were selected as the verification set, and KM analysis showed that the survival of the TCGA cohort, GSE72094, and GSE37745 middle- and high-risk groups was lower than that of the low-risk group (**Figure 5A**), and there were statistical differences (TCGA: p<0.001, GSE72094: p<0.001, GSE72094: p<0.001) 0.001, GSE37745: p=0.013). In addition, it was verified that the ROC curves performed well (**Figure 5B**). The results of Chi-square test showed that patients with later T stage and N stage had higher risk scores. The Sankey diagram showed that the survival status of patients with advanced stage was worse, and the proportion of dead patients was higher. We constructed a nomogram, in which T stage (p<0.001), N stage (p<0.001), age (p<0.001), riskscore (p<0.01) were all statistically significant (**Figures 5E, F**).

### Validation of OCIRGs

**Figure 6:**
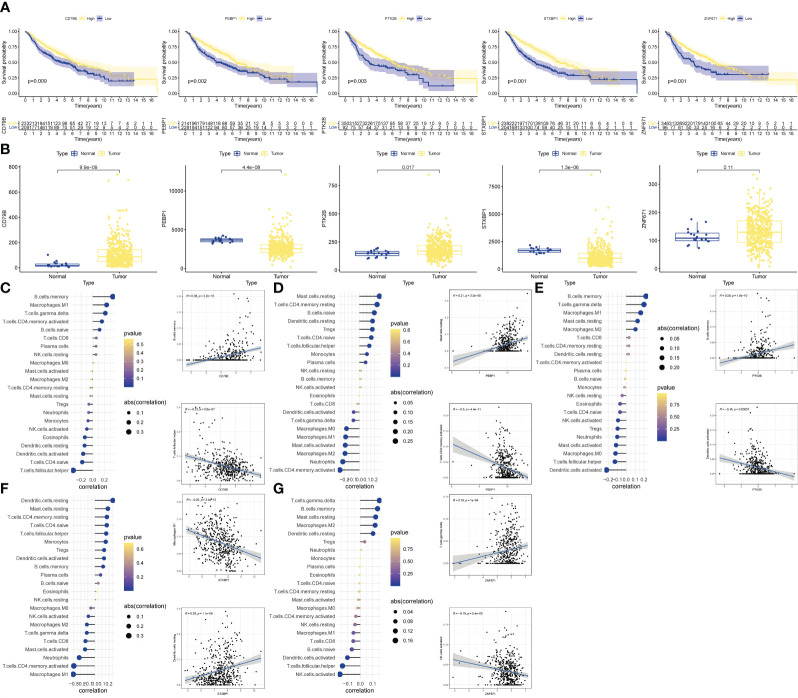
Validation of OCIRGS. **(A)** Kaplan-Meier curves of OS for high- and low-risk patients in the training sets and merged validation sets. **(B)** Expression changes of OCIRGS between normal and tumor tissues. **(C–G)** Associations between OCFRGs and immune-infiltrating levels. The color represents the significance. The greener, the more significant. The circle size represents the correlation coefficients.

We performed further analyzes on the genes that built the model. KM analysis showed that when OCIRG was highly expressed, the survival time of patients was shorter when the expression was lower (**Figure 6A**). Wilcoxon rank sum test showed that the expression of CD79B in OCIRG in LUAD was higher than that in normal tissues, and that of PEBP1, PTK2B, STXBP1, and ZNF671 was vice versa (**Figure 6B**). In terms of immune cell infiltration, the expression level of CD79B (**Figure 6C**) had the highest positive correlation with the infiltration level of B cells memory (R=0.38, p<0.001), and the highest negative correlation with Follicular helper T cell (R=-0.23, p <0.001). The expression level of PEBP1 (**Figure 6D**) had the highest positive correlation with resting mast cells (R=0.21, p<0.001), and the highest negative correlation with activated memory CD4 T cells (R=-0.3, p<0.001). The expression level of PTK2B (**Figure 6E**) had the highest positive correlation with memory B cells (R=0.29, p<0.001), and the highest negative correlation with activated dendritic cells (R=-0.16, p<0.001). The expression level of STXBP1 (**Figure 6F**) had the highest positive correlation with resting dendritic cells (R=0.28, p<0.001), and the highest negative correlation with Macrophages M1 (R=-0.32, p<0.001). The expression level of ZNF671 (**Figure 6G**) had the highest positive correlation with gamma delta T cells (R=0.18, p<0.001), and the highest negative correlation with activated NK cells (R=-0.19, p<0.001).

### Enrichment analysis associated with risk subtypes

**Figure 7:**
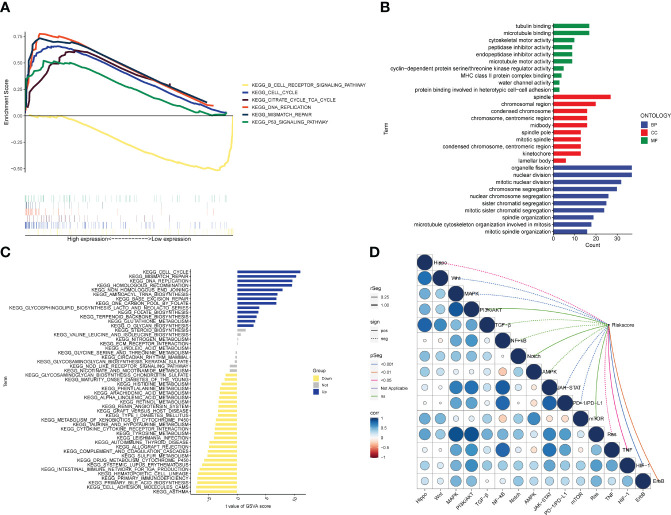
Biological functions. **(A)** Significant enriched pathways in the high- and low-risk groups. The extremum located in the left part indicates a positive association between risk scores and pathway activity, and vice versa. **(B)** Barplot graph for GO enrichment, with bar length representing the degree of enrichment and color representing the degree of difference. **(C)** There were significant differences in pathways between high and low risk groups. The blue bars represent a positive correlation between risk scores and pathway activity, and the opposite is true for yellow bars. **(D)** Correlations between Riskscore and important pathways in tumors.

According to the biological analysis of GSEA software, CELL CYCLE, CITRATE CYCLE TCA CYCLE, MISMATCH REPAIR, P53 SIGNALING PATHWAY were active in the high-risk group, and B CELL RECEPTOR SIGNALING was active in the low-risk patients (**Figure 7A**). GO functional analysis mainly involves chromosomes, mitosis and other functions (**Figure 7B**). The results of GSVA enrichment analysis showed that CELL CYCLE, DNA REPLTCATION, etc. were enriched in the high-risk group, and INTESTINAL IMMUNE NETWORK FOR IGA PRODUCTION, TYROSINE METABOLISM, ARACHIDONIC ACID METABOLISM, etc. were enriched in the low-risk group (**Figure 7C**). In addition, TNF, ErbB, HIF-1, JAK-STAT, Ras, PD-1/PD-L1, MAPK, Wnt, Hippo pathways, etc. were all correlated with the risk score (**Figure 7D**).

### Analysis of risk subtypes and tumor immunity

**Figure 8:**
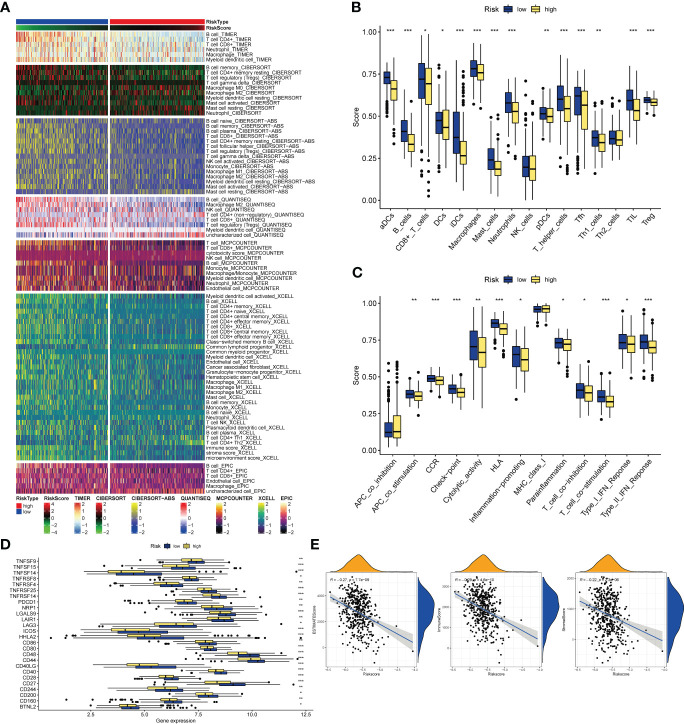
Immune-related analysis. **(A)** The relationships of risk and tumor immune-infiltrations according to the evidence from the TIMER database. **(B, C)** The differences of tumor infiltrating of 16 cell types and score of immune pathways between the risk groups by ssGSEA. The lines in the boxes represent the median values. The black dots represent outliers. Asterisks indicate significance. **(D)** The differences of expression level of immune checkpoints between the high-and-low-risk subtypes. The lines inside the boxes represent the median values, and the lines outsides the boxes indicate the 95% confidence interval. **(E)** The correlation between tumor purity and risk scores. The blue lines represent ftted lines, and the gray area represents the 95% confidence interval. The mountain graphs at the top and stuck to the right represent the density of distribution. *P<0.05, **P<0.01, ***P<0.001.

Analysis of the TIMER database showed that there were differences in the abundance of immune cell infiltration in the high- and low-risk groups, and the infiltration of CD8+ T cells, T cells, B cells, Neutrophil, and Myeloid dendritic cells in the low-risk group was significantly higher than that in the high-risk group (**Figure 8A**). As for the results of ssGSEA, it is divided into two parts: immune cells and immune function. The infiltration abundance of aDCs, B cells, CD8+ T cells, iDCs, Macrophages, Mast cells, Neutrophils, pDCs, T helper cells, Tfh, Th1_cells, TIL, T reg in the low-risk group was higher, only DCs in the high-risk group Infiltration levels were high in the risk group (**Figure 8B**), while in the low-risk group, APC co-stimulation, CCR, Check−point, Cytolytic activity, HLA, Inflammation−promoting, Parainflammation, T cell co−inhibition, T cell co− The levels of stimulation, Type I IFN Response, and Type II IFN Response were higher (**Figure 8C**), and all of them were statistically significant. In terms of immunotherapy, many immune checkpoints, including PD1 (PDCD1), were highly expressed in the low-risk group by Wilcox test analysis (**Figure 8D**). Subsequently, the correlation analysis between tumor purity and risk score showed that the immune scores ImmuneScore (R=-0.29) and StromalScore (R=-0.22) and the comprehensive score ESTIMATEScore (R=-0.27) were negatively correlated with the risk score, and there was a statistical academic significance (**Figure 8E**).

### Mutation landscape analysis

Analysis of gene mutation status in the high- and low-risk groups showed that the mutation frequencies of TP53, TTN, MUC16, and CSMD3 in the high-risk group were higher than those in the low-risk group (**Figures S6, 7**). Mutations include synonymous mutations and non-synonymous mutations, and the number of mutations of the three are positively correlated with the risk score, and there is a statistical difference (**Figures S8-10**).

### Overview of the scRNA-seq data generated from LUAD

**Figure 9:**
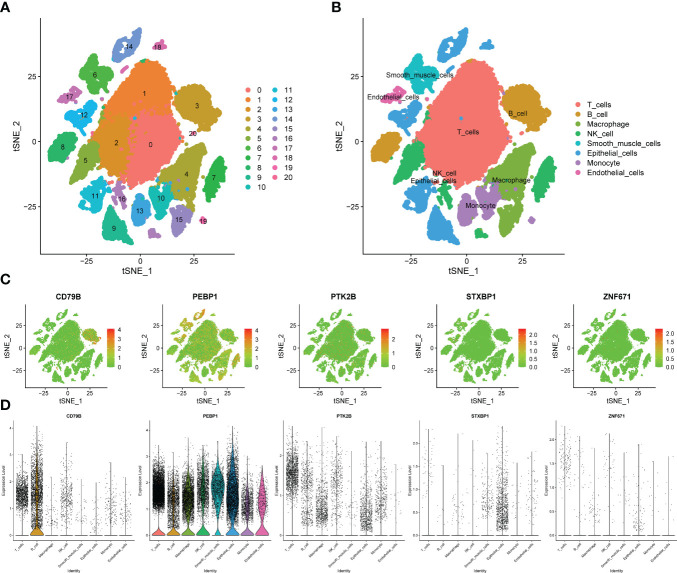
Verification of OCIRGs through sc-RNA seq. **(A, B)** tSNE plots of cells generated from LUAD tissue. The plots are colored by cell cluster, and the cells are clustered into 8 sub-clusters. Each dot represents a LUAD cell. **(C)** The expression of signature genes in LUAD visualized in tSNE. **(D)** Violin plots depicting the expression of signature genes in clusters of LUAD. The y axis shows the normalized read count. t-SNE:t-distributed stochastic neighbor embedding.

We obtained single-cell sequencing data for 12 samples from GSE168410. A total of 57223 cells were obtained by screening the total cells according to the intracellular gene features, the percentage of chromosome genes, etc. A total of 26 cell clusters were obtained (**Figure 9A**). We used tools to annotate the cell subsets, namely, T cells, NK cells, Macrophage, Epithelial cells, B cells, Smooth muscle cells, Monocyte, Endothelial cells (**Figure 9B**). To investigate the expression of marker genes in different cells, we visualized them with t-SNE and violin plots (**Figures 9C, D**). CD79B was highly expressed in B cells, while PEBP1 was highly expressed in various types of cells in the tumor microenvironment.

### Verification of OCIRGS expression

**Figure 10:**
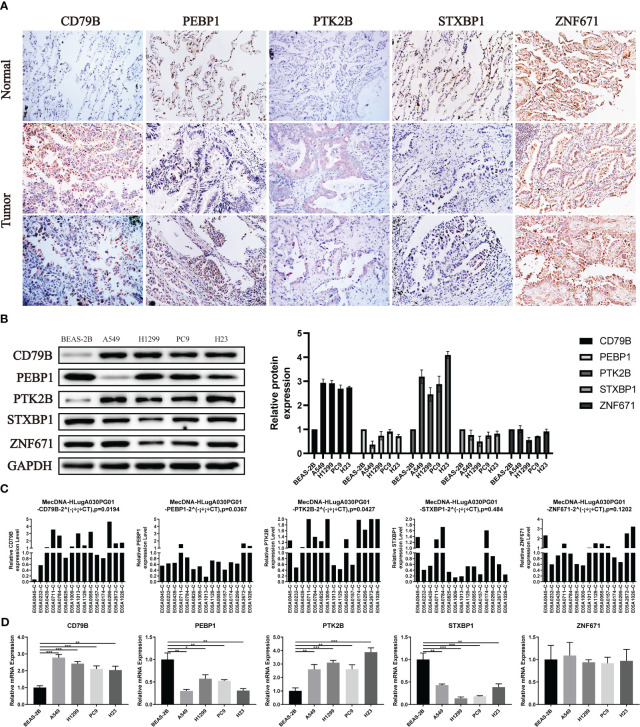
Verification of OCIRGS expression. **(A)** IHC verification of the expression level of OCIRGS in the LUAD tissue and surrounding tissue. **(B)** Western Bloting verifies the expression of OCIRGS in 1 normal cell strain and three types of LC cells. **(C, D)** PCR verification OCIRGS’s expression level. *P<0.05, **P<0.01, ***P<0.001.

For the expression of OCIRGs, we have conducted a series of experimental verification. The results of immunohistochemicals show that the expression of CD79B and PTK2B in lung adenocarcinoma is higher than tissue next to cancer. PEBP1 and STXBP1 are opposite, while Znf671 has no difference in expression next to tumors and cancer (**Figure 10A**). Then Western bloting is the same as IHC expressed in normal lung epithelial cell BEAS-2B and non-small cell lung cancer cells A549, H1229, PC9, and H23 (**Figure 10B**).

In order to further verify, we conducted PCR experiments using cells and tissues, and the results obtained are still the same as the above experiments (**Figures 10C, D**).

### Functional assessment of CD79B *in vitro*

**Figure 11:**
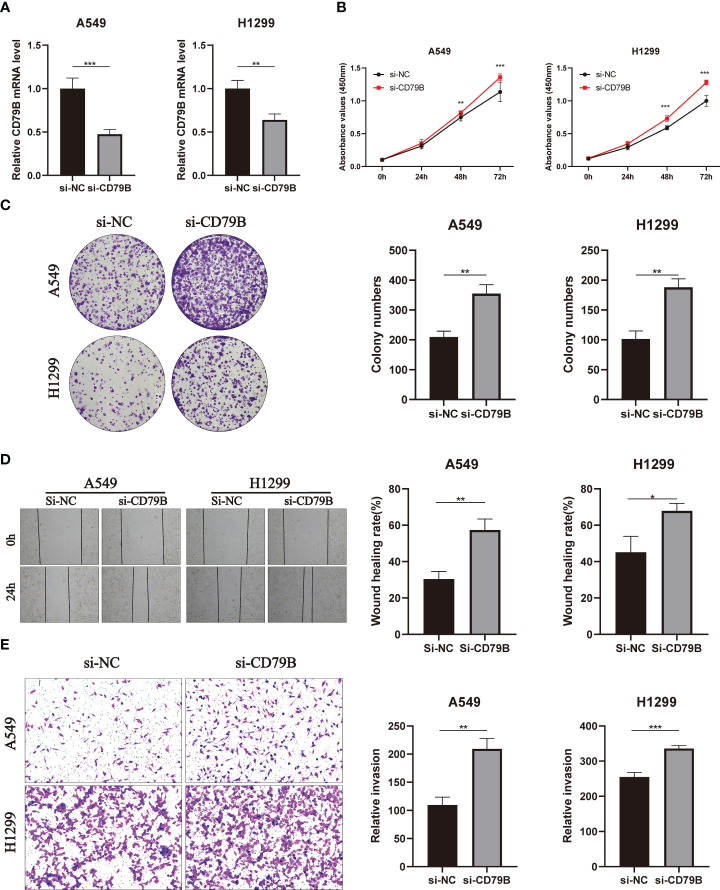
Validates the role of the key gene CD79B in lung cancer cell lines in vitro. **(A)** Knockdown of CD79B significantly reduced its expression in A549 and h1299 cell lines (**P<0.01, **P<0.001). **(B)** After CD79B knockdown in A549 and H1299 cell lines, the activity of lung adenocarcinoma cells was significantly enhanced (**P<0.01, *** (P<0.001). **(C)** Clonogenic assays showed a significant increase in the ability of A549 and H1299 cell lines to form colonies after CD79B knockdown (**P<0.01). **(D)** The si-NC group in the wound healing experiment of A549 and H1299 cell lines showed weaker migration ability than the si-CD79B group (*P<0.05, **P<0.01). **(E)** Knockdown of CD79B enhanced the invasion ability of A549 and H1299 cell lines (**P<0.01, *** (P<0.001).

In A549 and H1299 cell lines, the expression level of CD79B was significantly reduced after knockdown of CD79B mRNA (**Figure 11A**; ***P<0.001, **P<0.01). The activity of pancreatic cancer cells was also significantly reduced after CD79B knockdown in A549 and H1299 cell lines (**Figure 11B**; ***P<0.001, **P<0.001). Subsequently, colony formation analysis showed that the ability of A549 and H1299 cell lines to form colonies was significantly increased after CD79B knockdown (**Figure 11C**; **P<0.01, **P<0.01). The migration ability of A549 and H1299 cell lines in wound healing experiments was significantly increased after CD79B knockdown (**Figure 11D**; **P<0.01, *P<0.05). Knockdown of CD79B significantly reduced the invasive ability of A549 and H1299 cell lines (**Figure 11E**; **P<0.01, ***P<0.001) in transwell experiments.

## Discussion

In the past ten years, humans have made many breakthroughs in lung cancer research, and targeted drugs and immunotherapy drugs have been continuously updated. The multidisciplinary treatment model and new therapeutic drugs have brought new hope to the treatment of lung cancer (25). However, the survival time of lung cancer has not improved significantly. The high mortality rate of lung cancer is still a difficult problem to solve. We still need to explore new robust markers to guide clinical treatment decisions. In 2022, cuproptosis was first proposed as a new cell death mode related to TCA cycle (26, 27). The emergence of cuproptosis has brought a new research direction to the treatment of tumors, and immunity is undoubtedly the focus of tumor research, and there is a certain relationship between the two. In addition, previous studies have not analyzed the two together. To address the shortcomings of previous studies, this study innovatively associates cuproptosis with immune function. By analyzing the CIRG-related prognosis model using bioinformatics methods, the study evaluates the value of prognosis and immune therapy for LUAD patients. The results show that the prognosis model related to copper death and immune function can accurately predict the prognosis and level of lung cancer immune cell infiltration in LUAD patients. In addition, this model can guide clinicians to make personalized treatment decisions and provide a basis for the study of lung adenocarcinoma and tumor microenvironment.

In this study, we screened and analyzed 5 OCIRGs (CD79B, PEBP1, PTK2B, STXBP1, ZNF671), and further analyzed the prognosis model constructed for OCIRGs. This includes analysis of the tumor immune microenvironment, enriched functional pathways in the risk subtypes, and analysis of OCIRGs in single-cell sequencing.

CD79b Molecule (CD79B) is a critical component of the B cell receptor complex, which can recognize antigens and affect the growth and differentiation of B cells by activating an internal signaling pathway. CD79B, along with CD79A, IgH, and IgL chain molecules, forms a complex that participates in the activation of the B cell receptor (BCR) signaling pathway (28, 29). CD79B plays different roles in different lymphoma types. In mucosa-associated lymphoid (MALT) lymphoma, the chronic activation of the CD79B/BTK pathway enhances the proliferation of lymphoma cells (30). In diffuse large B-cell lymphoma, it increases cell proliferation, survival, and invasion capabilities and is widely mutated (30, 31). Polatuzumab (32, 33), an antibody drug conjugates(ADC) antibody drug targeting CD79B, binds to the CD20 antigen, inhibiting B cell growth and proliferation in the CD79B region, and has been proven to have therapeutic effects in specific types of lymphomas, such as DLBCL and EMZL refractory or relapsed cases. Therefore, CD79B may become a biomarker for lymphoma and provide valuable assistance for early prediction, diagnosis, and treatment of lymphoma. Currently, there is no in-depth study of the biological mechanism of CD79B in lung cancer. This study’s results show that CD79B is highly expressed in LUAD and is associated with a good prognosis. In addition, the expression level of CD79B is positively correlated with the abundance of memory B cell infiltration, confirming their close association in LUAD. As a tumor suppressor gene, when the expression of CD79B decreases, the vitality, proliferation, migration, and invasion capabilities of non-small cell lung cancer cells increase, indicating that CD79B may have some biological significance in tumor treatment and is worth studying further. Phosphatidylethanolamine Binding Protein 1 (PEBP1) is a critical protein in various biological processes. Studies have shown that PEBP1 is closely related to the occurrence, metastasis, and prognosis of various cancers. In non-small cell lung cancer (NSCLC), PEBP1 has been proven to be one of the key proteins regulating the proliferation and metastasis of lung cancer cells. Its function mainly involves affecting the tumor’s radiosensitivity (34), regulating JAK/STAT3 (35), inhibiting NF-κB (36), controlling the TGF-β of cells and inhibiting (36) MAPK/ERK signaling pathways (37). In NSCLC, the expression level of PEBP1 is closely related to the severity of lung cancer and patients’ prognosis. Overexpression of PEBP1 can significantly inhibit the proliferation, migration and invasion of lung cancer, reduce tumor size, and the probability of distant metastasis (38). In addition to lung cancer, PEBP1 plays a regulatory role in multiple cancers such as breast cancer (39, 40), prostate cancer (41) and ovarian cancer (42). Downregulation of PEBP1 expression can promote tumor development and metastasis, while overexpression of PEBP1 can inhibit tumor metastasis and is of significant importance for patients’ prognosis and treatment. In immune infiltration analysis, the expression of PEBP1 is positively correlated with quiescent mast cells, which may be related to PEBP1’s negative regulation of mast cell activation. Protein Tyrosine Kinase 2 Beta (PTK2B) is a tyrosine kinase protein that plays a very important role in cell growth, apoptosis, and signal transduction (43). Recent studies have shown that PTK2B plays an important role in many cancers. In non-small cell lung cancer (NSCLC), PTK2B activation can cause many cellular biological processes, including proliferation, differentiation, migration, invasion, and apoptosis (44–46). In addition to lung cancer, PTK2B also plays different roles in other types of cancers. In breast cancer (47), PTK2B overexpression is associated with tumor growth and recurrence, playing a role in countering BMP, a protein that controls cell growth and differentiation. In liver cancer (48, 49), PTK2B expression is associated with tumor invasion behavior and poor prognosis. Database analysis has also shown high expression of PTK2B in lung adenocarcinoma tissue, which is correlated with a good prognosis. Immune infiltration analysis shows that PTK2B expression is positively correlated with the abundance of memory B cell infiltration. Studies have also shown that PTK2B phosphorylation is critical for TLR9-driven B cell proliferation and differentiation, thereby affecting memory B cell infiltration abundance. Syntaxin Binding Protein 1 (STXBP1) is an important protein that mainly participates in regulating the fusion of intracellular granule membranes and plays a role in the cell’s killing function and immune regulation processes (50). It is one member of the Munc protein family, widely present in eukaryotic cells, and participates in the fusion and release processes of granule membranes (50). STXBP1 plays a crucial role in tumor cell killing and granule cell granule membrane fusion. In addition, abnormal expression of STXBP1 also plays an important role in many diseases. It has been reported that STXBP1 is highly expressed in LUAD (51), which is different from this study’s results. This study analyzed and experimentally verified the LUAD data from TCGA and GEO databases, and the results showed that STXBP1 was lowly expressed in tumors. Taken together, it may be due to the different sample sizes selected by the two studies or the different treatment methods included in the samples. Zinc Finger Protein 671 (ZNF671) is a zinc finger transcription factor gene that belongs to the KRAB-ZF transcription family (52). During cell development, this gene participates in many important biological processes by binding to specific sequences on DNA. Previous studies have shown that ZNF671 is a tumor suppressor gene that is silenced by epigenetic modifications (53). This gene has different functional states in different cancer types, including apoptosis, cell cycle, DNA damage and repair, anaerobic conditions, inflammation, invasion and metastasis, proliferation, and stemness. Recent studies have found that the expression level of ZNF671 in non-small cell lung cancer is associated with an increased risk of disease progression and metastasis (54). ZNF671 can suppress the growth and spread of lung cancer by inhibiting the Wnt/β-catenin signaling pathway (54). The Wnt/β-catenin signaling pathway is a complex cellular signaling pathway that participates in regulating cell fate, proliferation, differentiation, and cell polarity, among other processes. The pathway is closely related to the occurrence and development of lung cancer. ZNF671 plays an important role in regulating this signaling pathway, and its regulatory mechanism may involve the participation of multiple molecules and signaling pathways. ZNF671 expression is significantly correlated with the prognosis of LUAD, but its expression does not differ significantly between normal and tumor tissues. The results on the training set are not statistically significant. Then we analyzed the TCGA LUAD dataset, which showed that ZNF671 was downregulated in tumor tissues compared to normal tissues, with statistical differences, but the difference was not significant (p=0.04). In addition, both cell-level and tissue-level experimental results were not significant. In conclusion, there should be no difference in the expression of ZNF671 between normal and tumor tissues.The proportional hazards regression model in this study was also constructed based on the above five OCIRGs. Through functional analysis, it can be found that cell cycle, citrate cycle tca cycle, mismatch repair, p53 signaling pathway, etc. are active in the high-risk group, and only B cell receptor signaling_pathway plays a greater role in the low-risk group. Among them, cell cycle is mainly related to tumor cell proliferation (55, 56), and enrichment in high-risk groups will increase risk factors. The TCA cycle is the hub of energy metabolism (57, 58) and is directly related to cuproptosis. In addition, when the expression of tumor suppressor genes and oncogenes of cancer cells is out of regulation, they will rely heavily on TCA cycle for energy metabolism, and the maintenance of TCA cycle can promote cancer metastasis (59). DNA mismatch repair (MMR) maintains genome stability, as gene mutations can promote cancer initiation and progression (60, 61). In addition, the risk score was positively correlated with scores of pathways such as TNF, ErbB, and HIF-1, and negatively correlated with scores of signaling pathways such as JAK-STAT, Ras, PD-1/PD-L1, MAPK, Wnt, and Hippo. The obtained results can also be used in subsequent studies of models and pathways.

Through the different algorithms of TIMER and ssGSEA, consistent results were obtained, and the infiltration abundance of most immune cells in the high-risk group was lower. This also confirmed the poor prognosis of the high-risk group from the side. Immune checkpoints and TMB are important tumor immunotherapy markers. The results obtained in this study show that the expression of immune checkpoints in the high-risk group is lower than that in the low-risk group, and most importantly, PD1, a clinically applied marker, is also included. The opposite results were obtained for TMB, with higher TMB in the high-risk group, and a high TMB indicating that patients responded better to immunotherapy. Considering that the human body is a huge system with complex regulatory mechanisms, further research is needed.

scRNA-seq analysis showed that PEBP1 was highly expressed in various cells in the tumor microenvironment, including T cells, NK cells, Macrophage, Epithelial cells, B cells, Smooth muscle cells, Monocyte, and Endothelial cells. Therefore, we speculate that there is an important relationship between the expression of PEBP1 and immune activation and immune microenvironment. On the other hand, CD79B is highly expressed in B cells, which is also consistent with previous reports.

## Conclusion

In this study, we screened genes in LUAD using bioinformatics and machine learning methods, and picked out 5 OCIRGs to build a prognosis model. Using this model, the prognosis and abundance of immune infiltrates in LUAD patients can be accurately predicted. Based on this model, it can guide clinicians to make personalized treatment decisions, and also provides an important basis for the study of LUAD and tumor microenvironment.

## Data availability statement

The datasets presented in this study can be found in online repositories. The names of the repository/repositories and accession number(s) can be found in the article/**Supplementary Material**.

## Author contributions

ZY, FZ, and YeW designed this study. WZ, XM, LL, and HQ completed the bioinformatics analysis. WZ completed the writing of the manuscript. KY, YN, WZ, HQ, and CZ were tested *in vitro*. All authors contributed to the article and approved the submitted version.
