# Supplementary figures and images for: Identification of cuproptosis and immune-related gene prognostic signature in lung adenocarcinoma
Source: Front Immunol. 2023 Aug 9;14:1179742. doi: 10.3389/fimmu.2023.1179742 (PMC10445162; doi:10.3389/fimmu.2023.1179742)

1

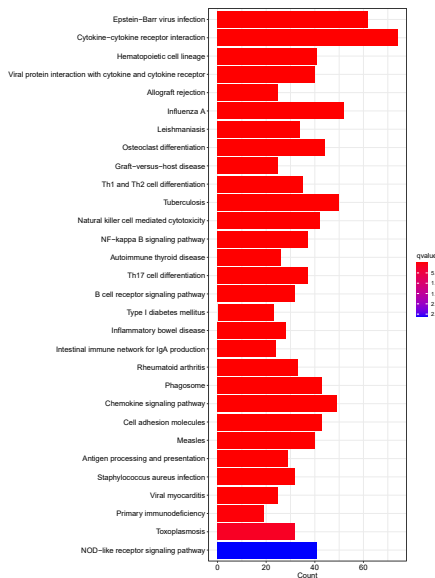

2

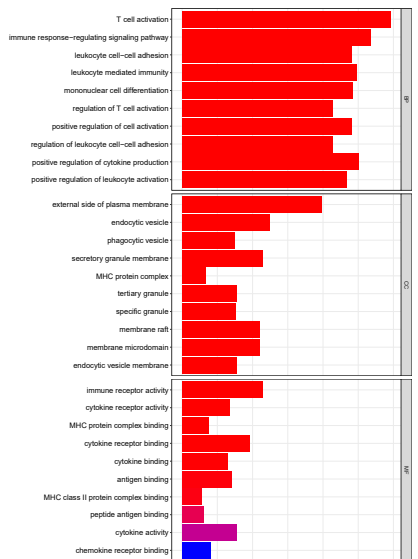

3

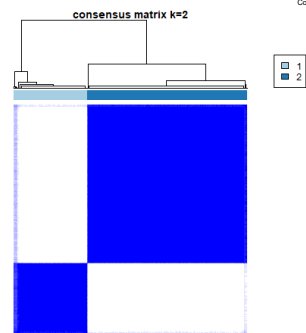

4

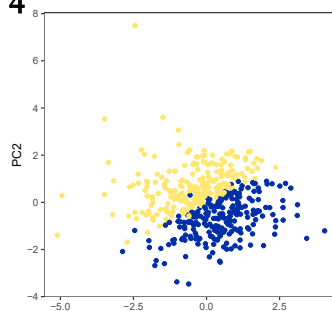

5

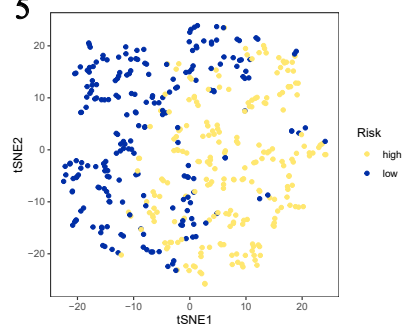

6

Altered in 223 (95.3%) of 234 samples.

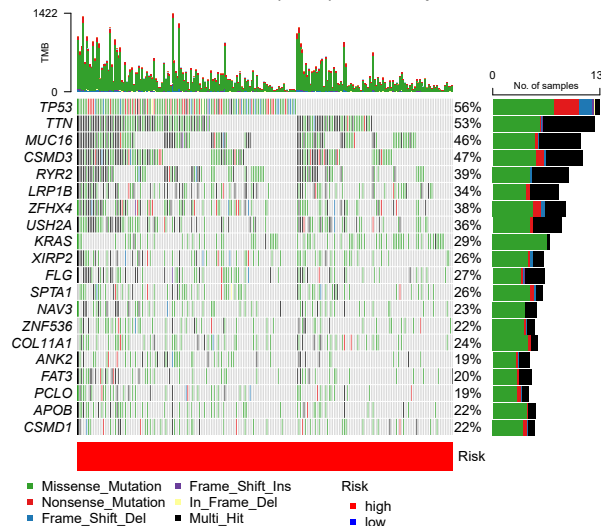

7

Altered in 194 (86.61%) of 224 samples.

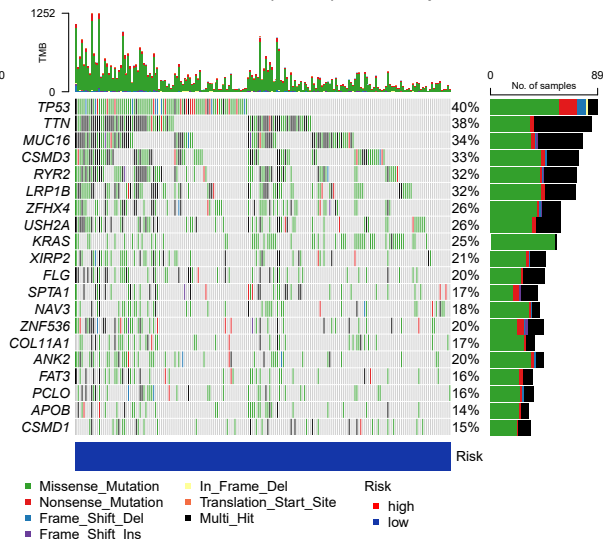

8

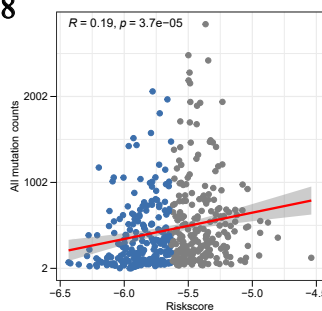

9

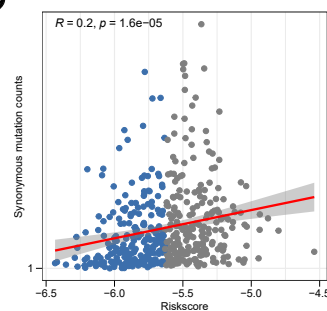

10

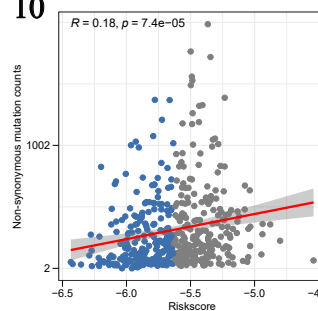

Supplement: Supplementary file 1 [file DataSheet_1.pdf]

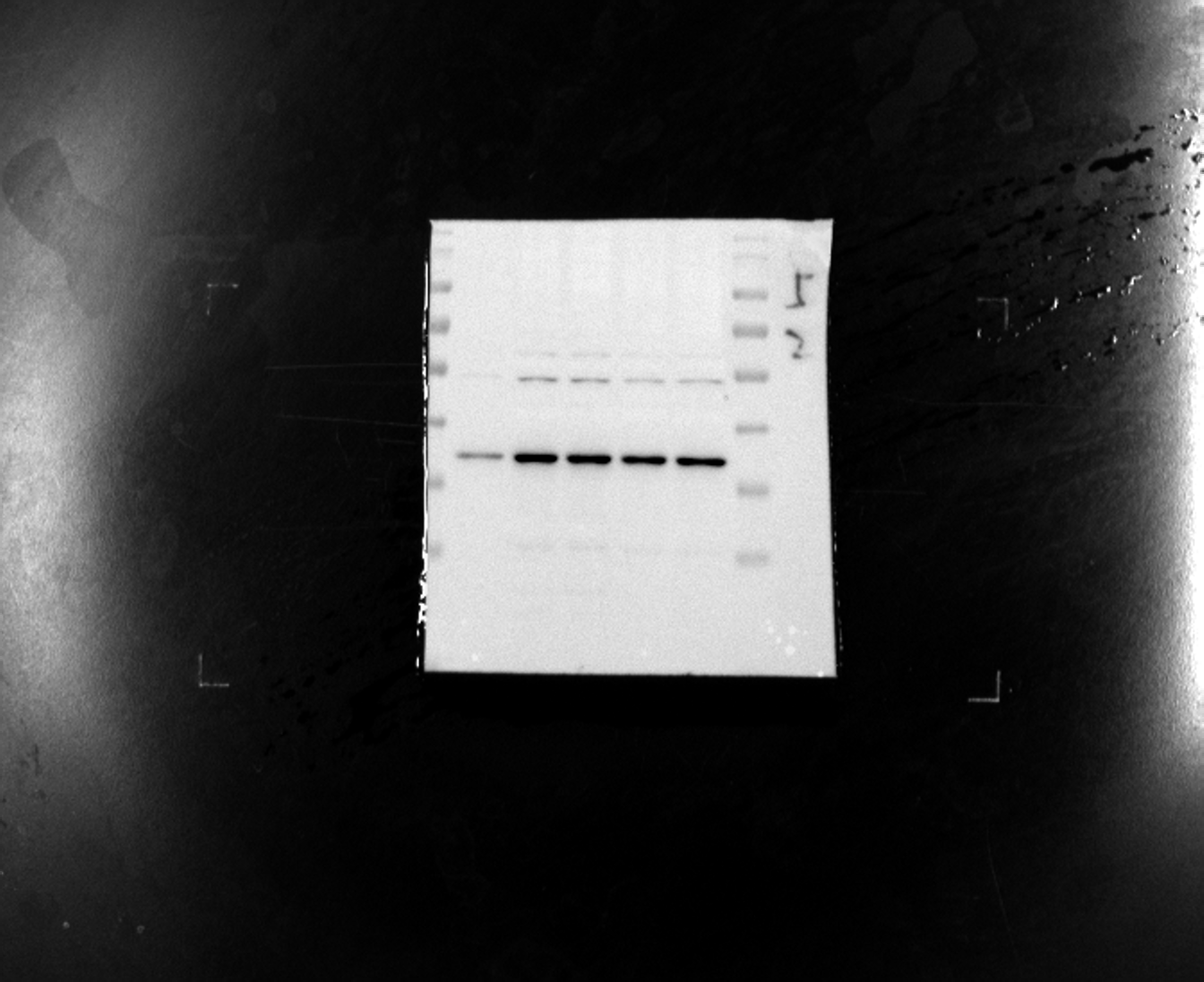

Supplement: Supplementary file 2 [file DataSheet_2.zip › CD79B(26-40)-1..Tif]

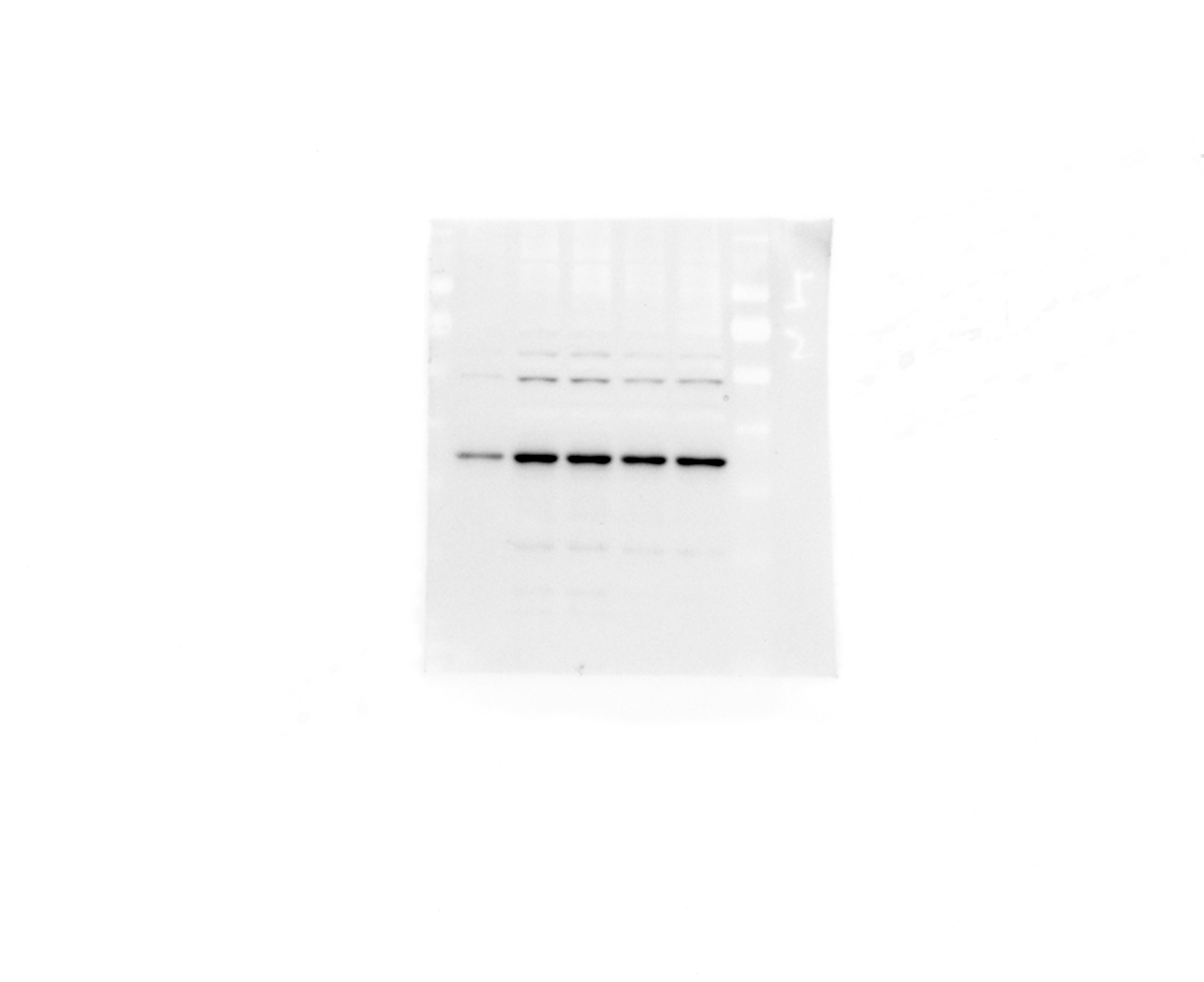

Supplement: Supplementary file 2 [file DataSheet_2.zip › CD79B(26-40)-1.Tif]

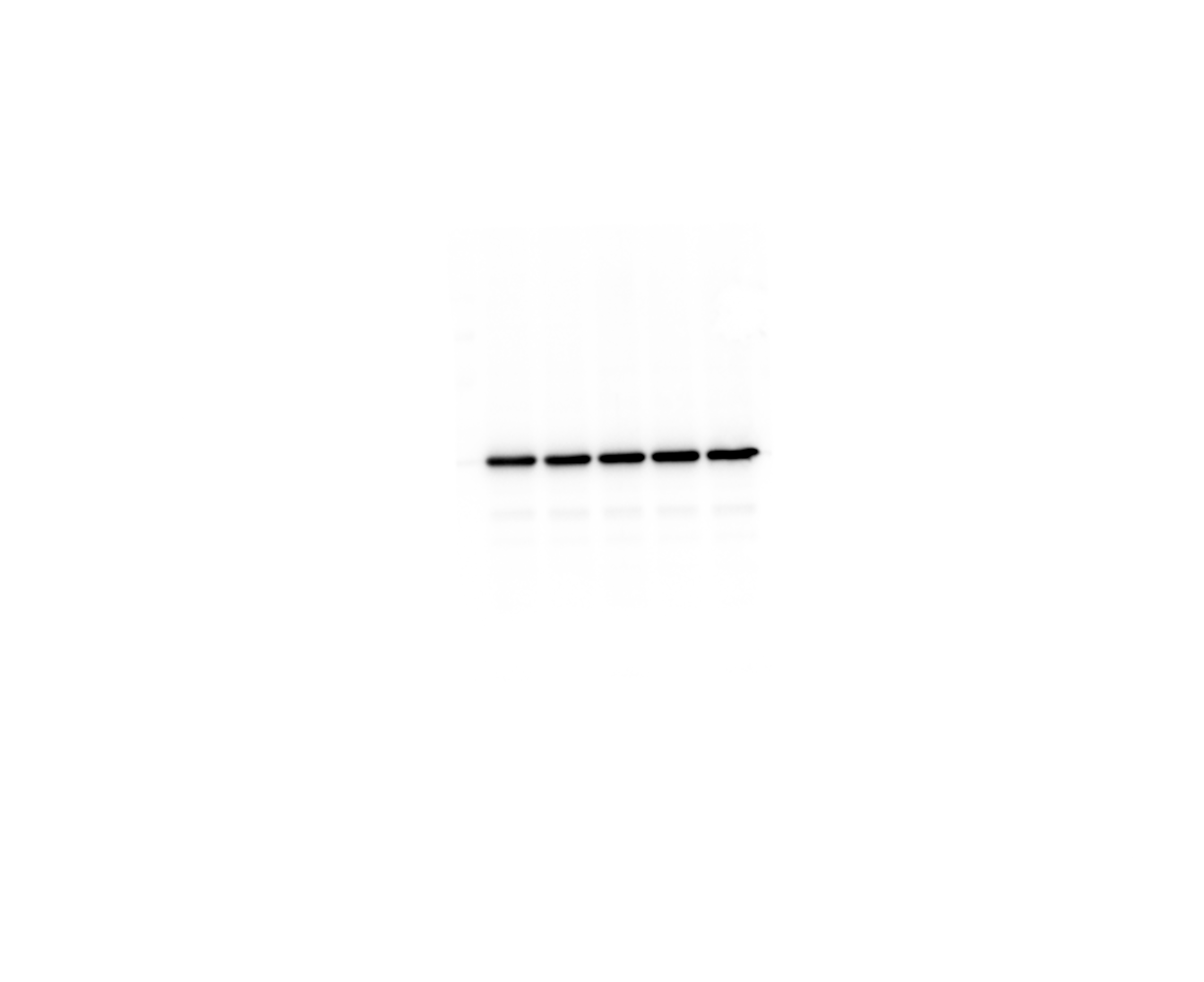

Supplement: Supplementary file 2 [file DataSheet_2.zip › GAPDH-1.Tif]

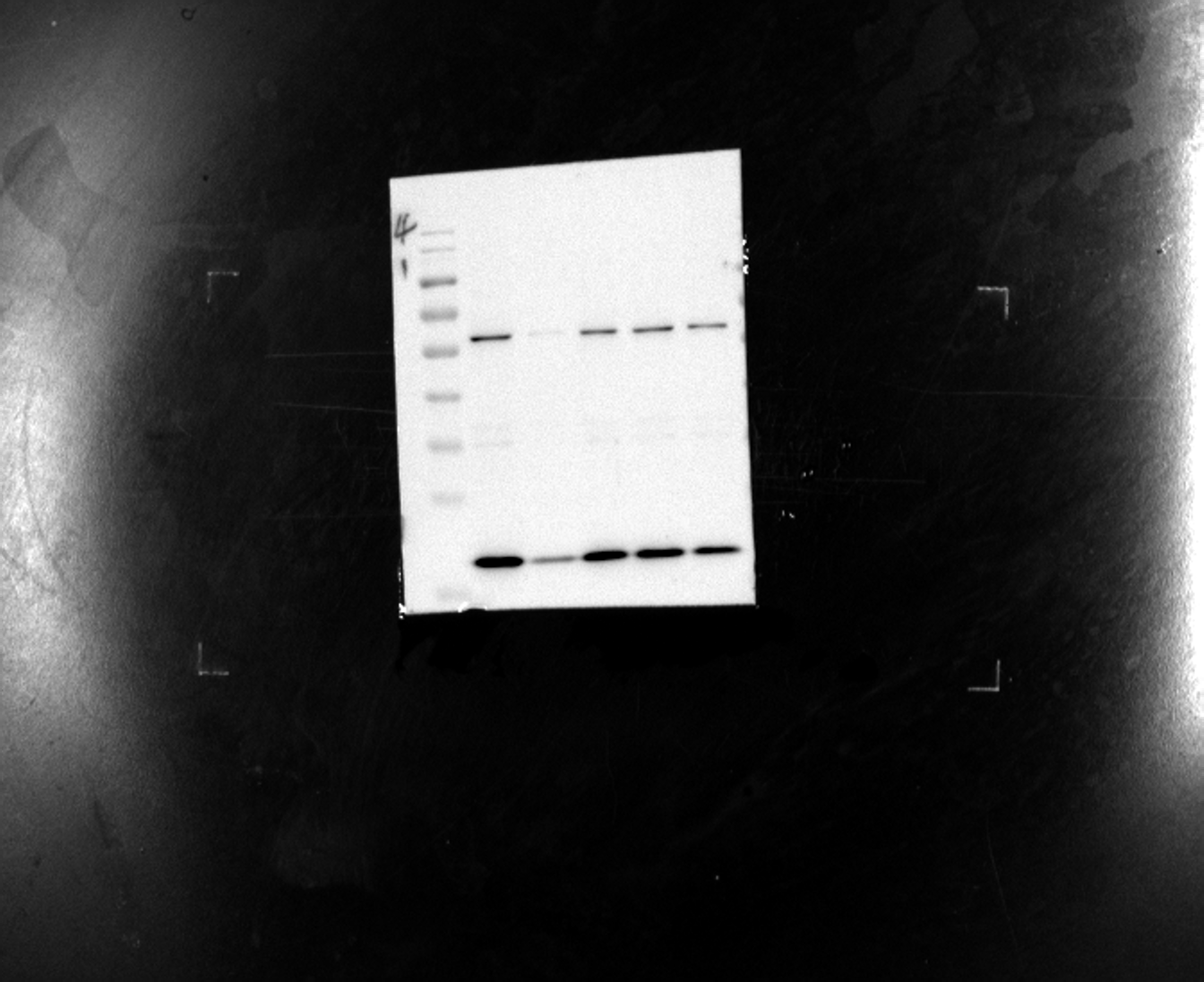

Supplement: Supplementary file 2 [file DataSheet_2.zip › PEBP1..Tif]

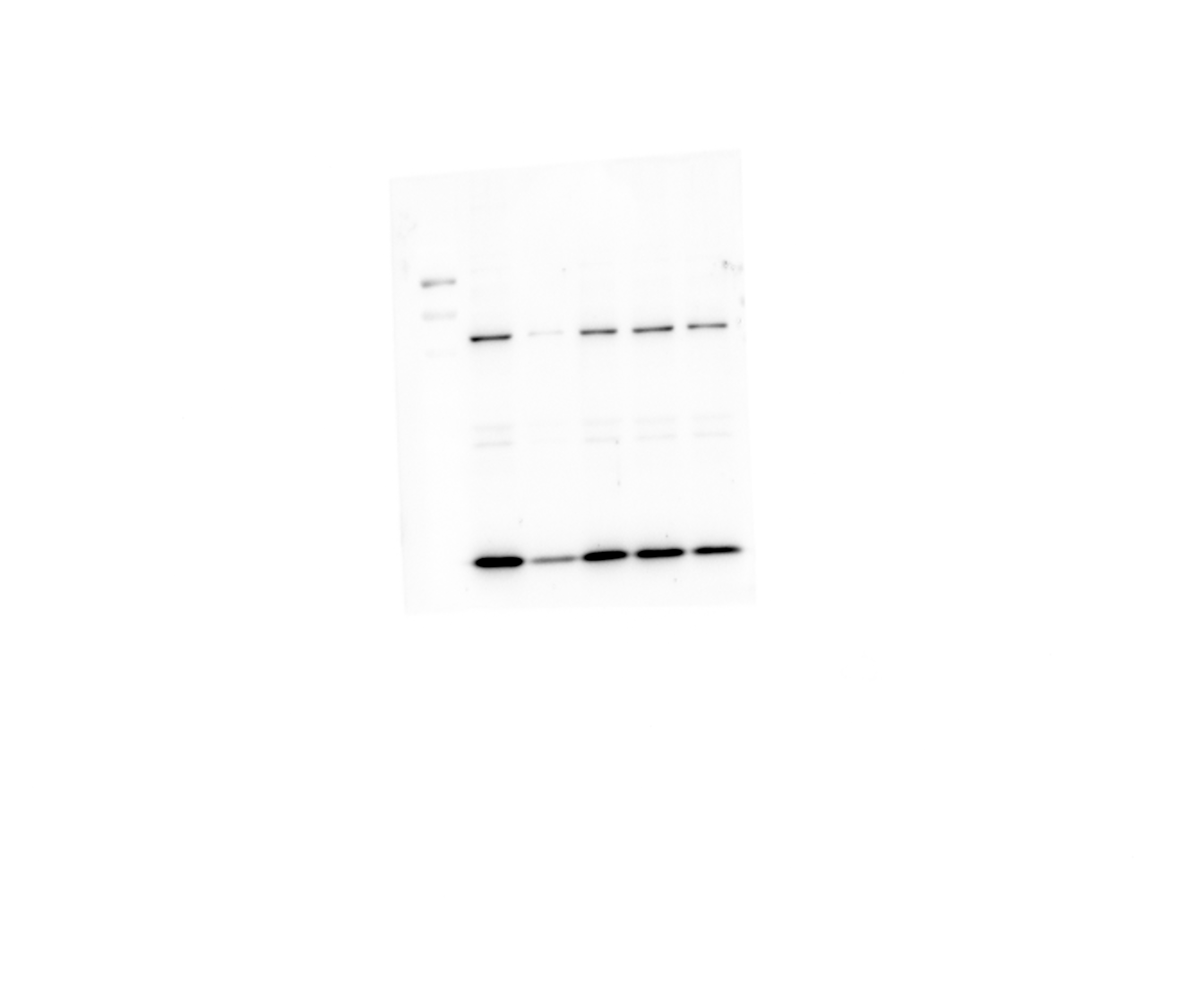

Supplement: Supplementary file 2 [file DataSheet_2.zip › PEBP1.Tif]

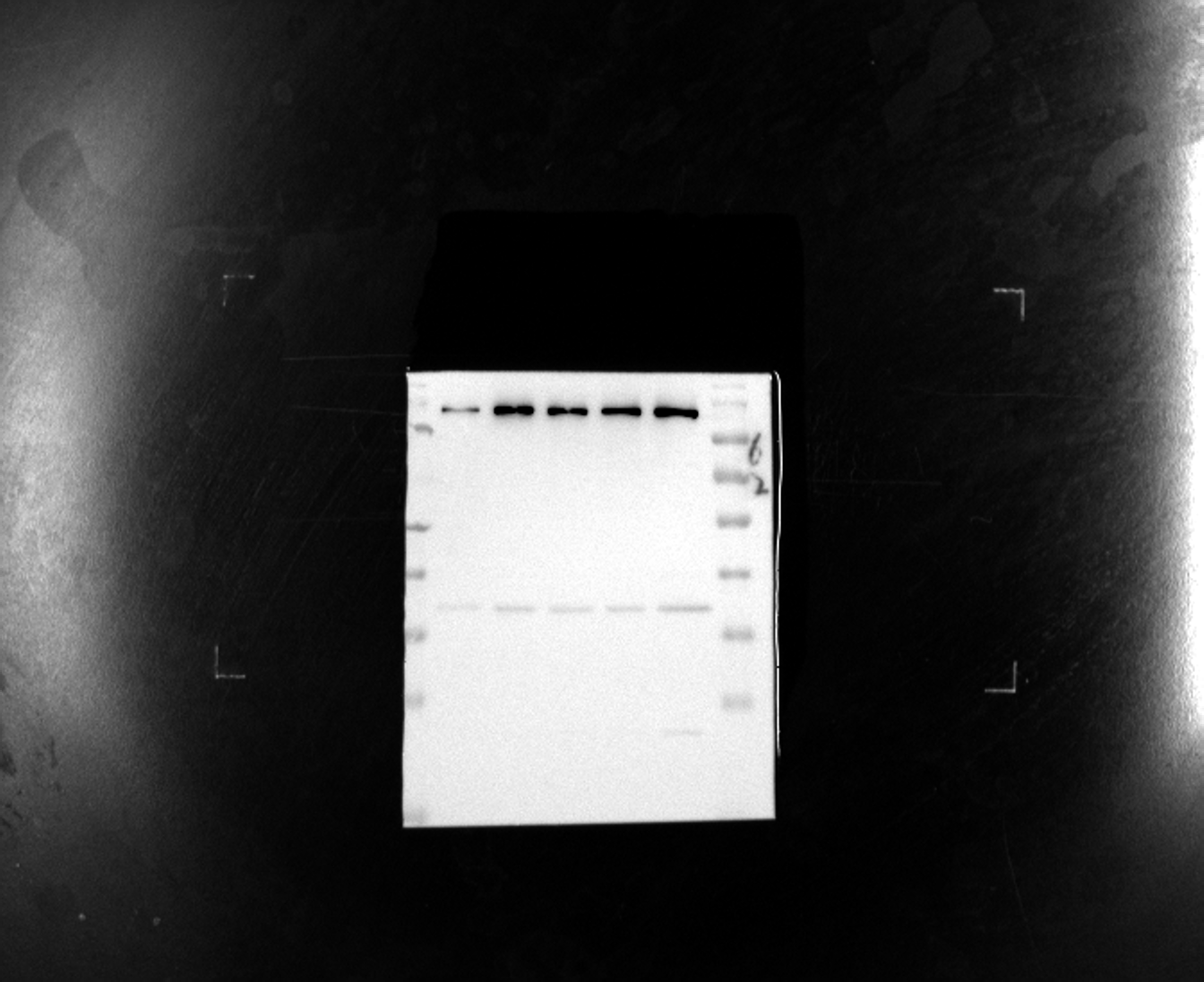

Supplement: Supplementary file 2 [file DataSheet_2.zip › PKT2B(116)-1..Tif]

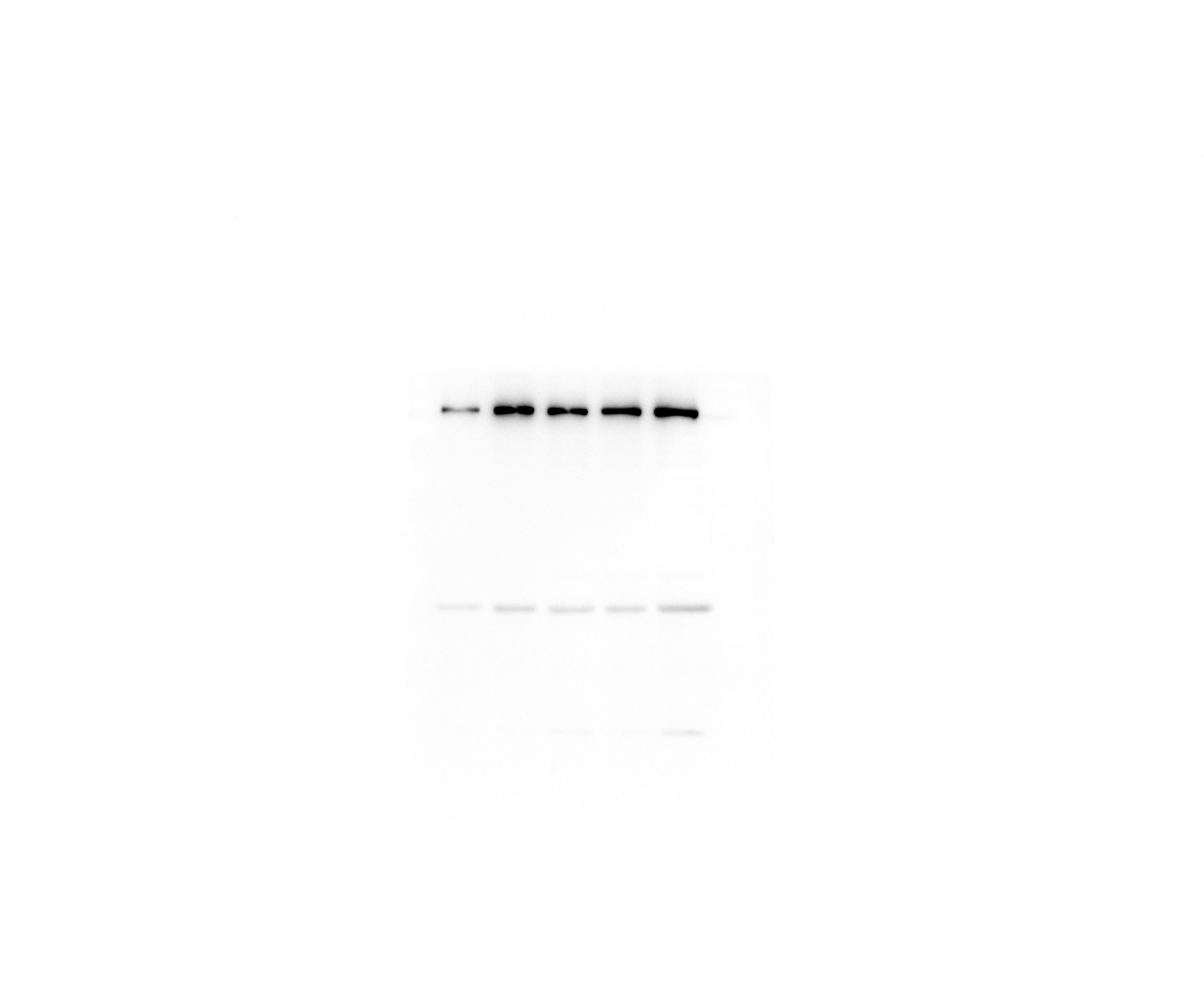

Supplement: Supplementary file 2 [file DataSheet_2.zip › PKT2B(116)-1.Tif]

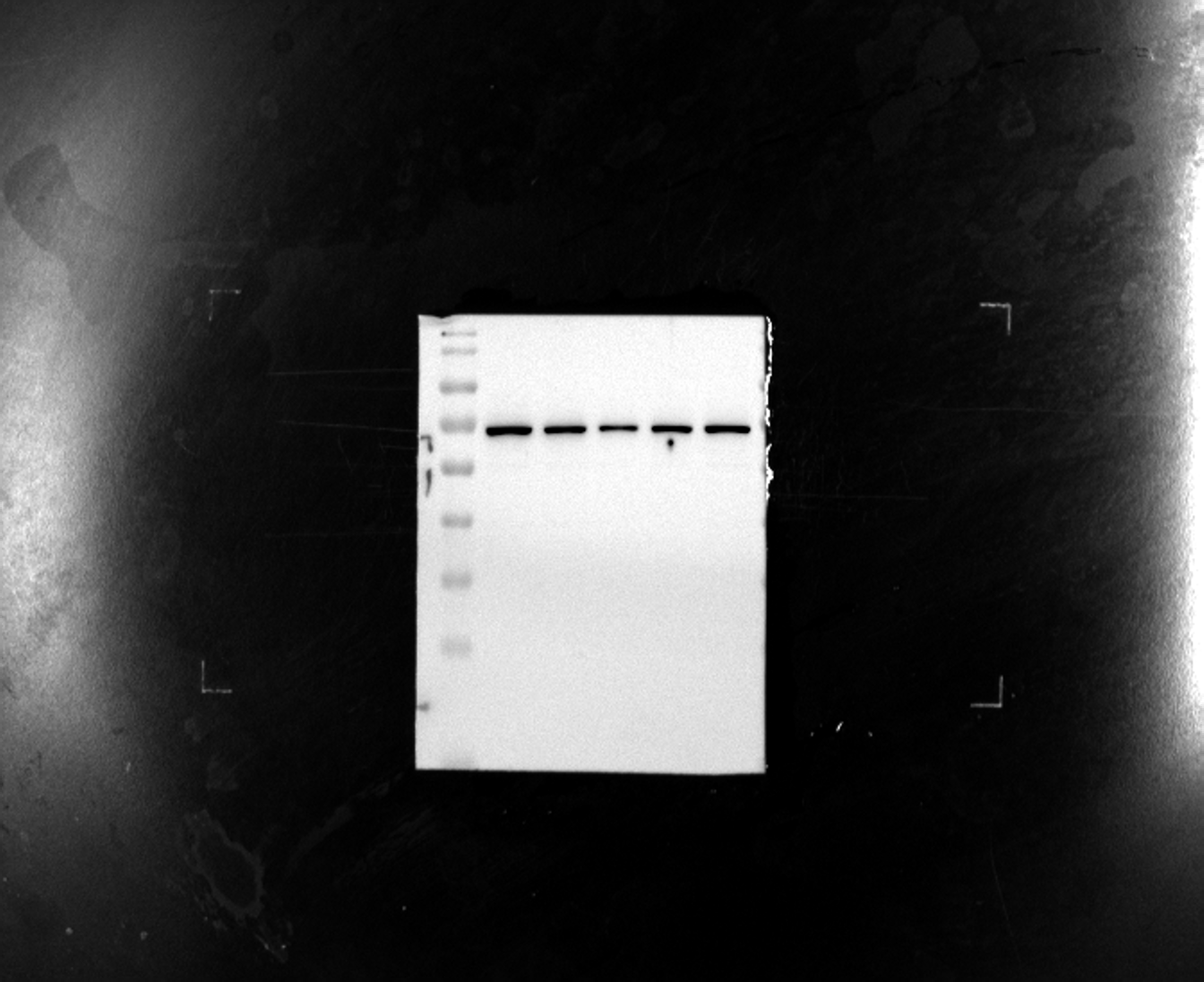

Supplement: Supplementary file 2 [file DataSheet_2.zip › STXBP1(68)-1..Tif]

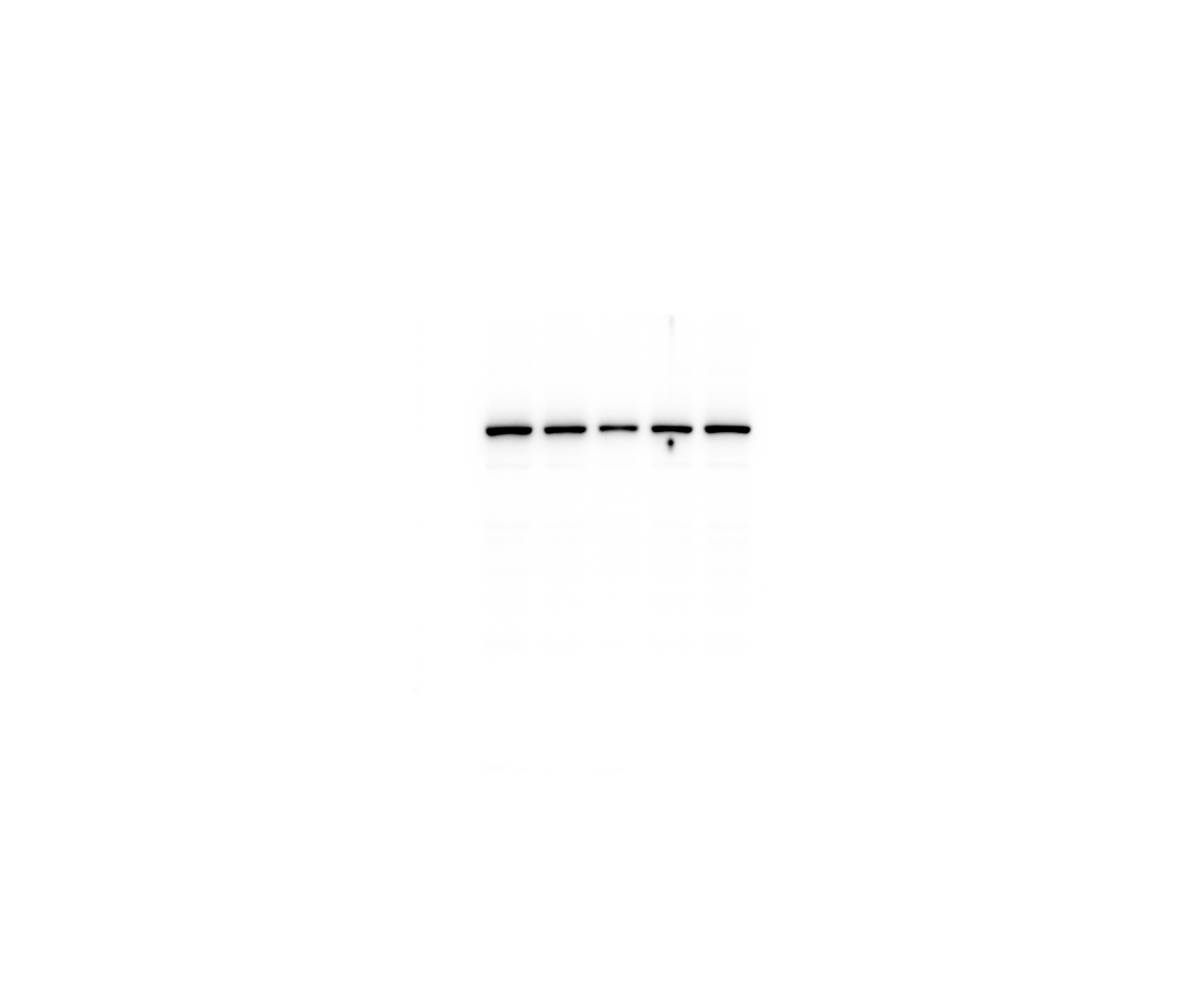

Supplement: Supplementary file 2 [file DataSheet_2.zip › STXBP1(68)-1.Tif]

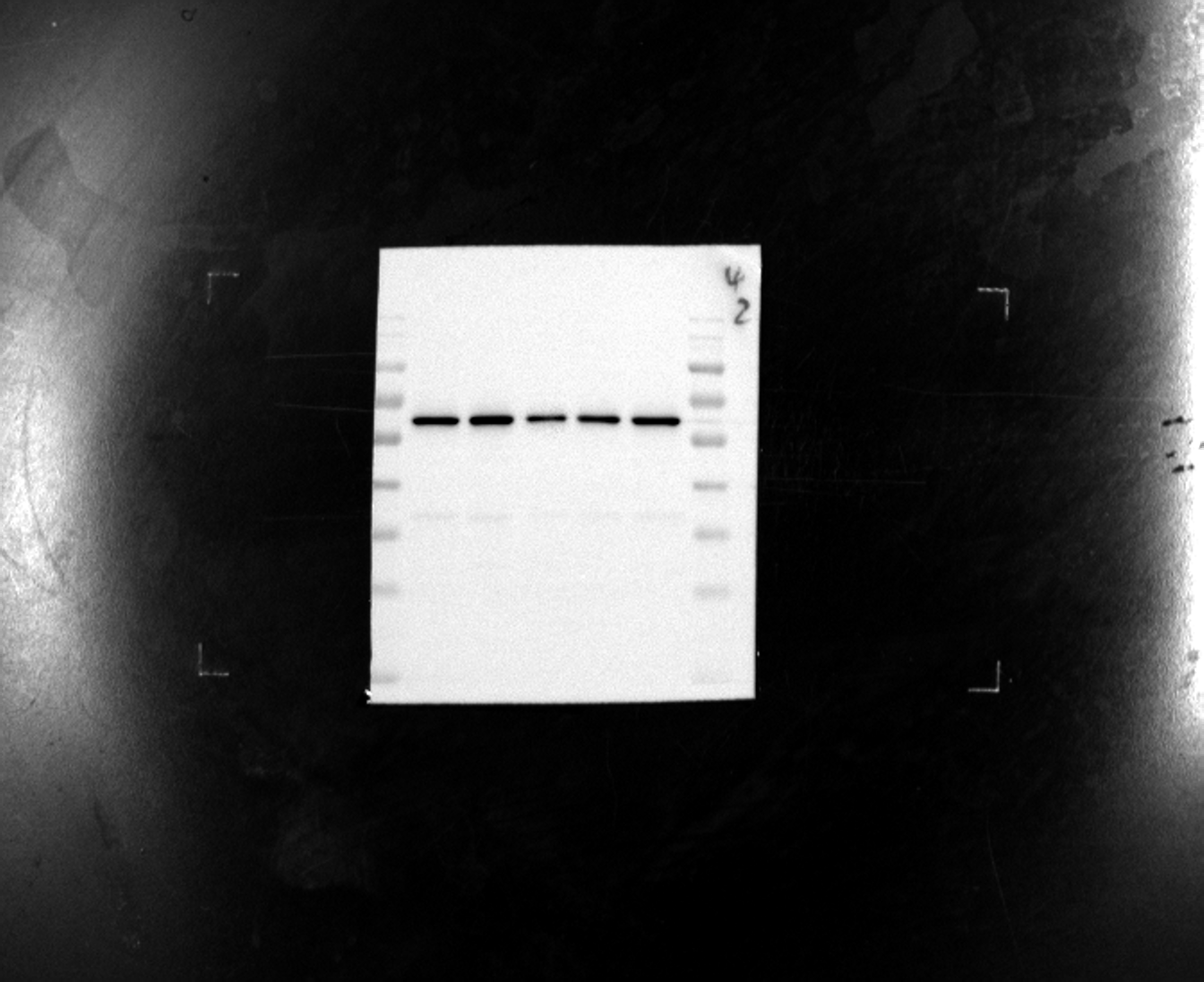

Supplement: Supplementary file 2 [file DataSheet_2.zip › ZNF671..Tif]

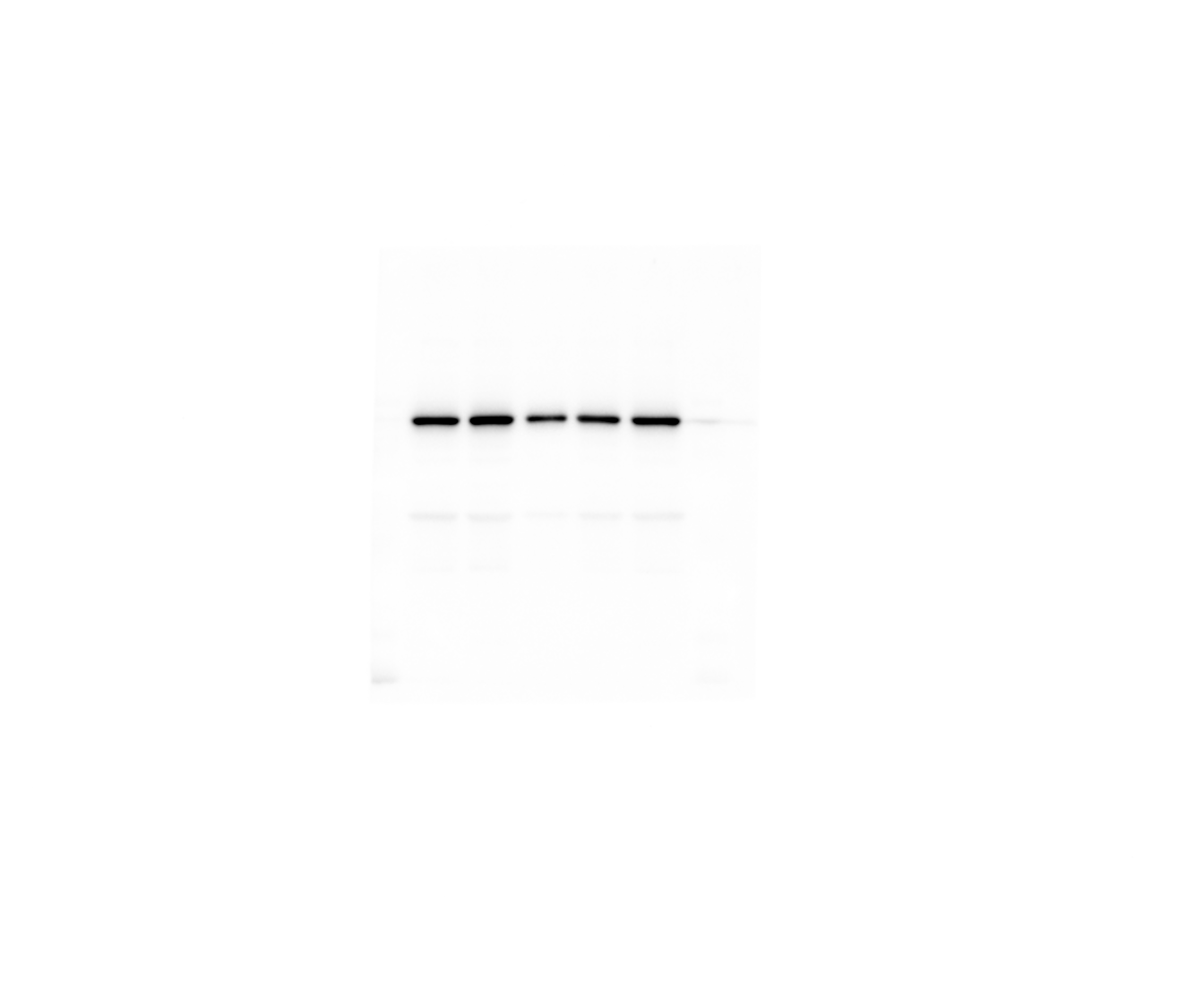

Supplement: Supplementary file 2 [file DataSheet_2.zip › ZNF671.Tif]
